# Intermolecular interactions underlie protein/peptide phase separation irrespective of sequence and structure at crowded milieu

**DOI:** 10.1038/s41467-023-41864-9

**Published:** 2023-10-04

**Authors:** Manisha Poudyal, Komal Patel, Laxmikant Gadhe, Ajay Singh Sawner, Pradeep Kadu, Debalina Datta, Semanti Mukherjee, Soumik Ray, Ambuja Navalkar, Siddhartha Maiti, Debdeep Chatterjee, Jyoti Devi, Riya Bera, Nitisha Gahlot, Jennifer Joseph, Ranjith Padinhateeri, Samir K. Maji

**Affiliations:** 1 Department of Biosciences and Bioengineering, IIT Bombay, Powai, Mumbai, 400076 India; 2 Sunita Sanghi Centre of Aging and Neurodegenerative Diseases, IIT Bombay, Powai, Mumbai, 400076 India; 3 Present Address: Department of Bioengineering, VIT Bhopal University, Bhopal-Indore Highway, Kothrikalan, Sehore, Madhya Pradesh 466114 India

**Keywords:** Biophysical chemistry, Intrinsically disordered proteins, Structural biology

## Abstract

Liquid-liquid phase separation (LLPS) has emerged as a crucial biological phenomenon underlying the sequestration of macromolecules (such as proteins and nucleic acids) into membraneless organelles in cells. Unstructured and intrinsically disordered domains are known to facilitate multivalent interactions driving protein LLPS. We hypothesized that LLPS could be an intrinsic property of proteins/polypeptides but with distinct phase regimes irrespective of their sequence and structure. To examine this, we studied many (a total of 23) proteins/polypeptides with different structures and sequences for LLPS study in the presence and absence of molecular crowder, polyethylene glycol (PEG-8000). We showed that all proteins and even highly charged polypeptides (under study) can undergo liquid condensate formation, however with different phase regimes and intermolecular interactions. We further demonstrated that electrostatic, hydrophobic, and H-bonding or a combination of such intermolecular interactions plays a crucial role in individual protein/peptide LLPS.

## Abstract (web summary)

In this work, the authors report that proteins/polypeptides with diverse sequences and structures can form liquid condensates, however with varying phase regimes and different modes of intermolecular interactions, spanning from electrostatic, hydrophobic and H-bond.

## Introduction

Liquid-liquid phase separation (LLPS) of biomolecules (proteins/nucleic acids) is now well-established as a ubiquitous phenomenon for the formation of membraneless organelles^1–6^. These phase separated, condensed compartments not only help in various cellular functionality^7–9^; but they are also useful for macromolecular sequestration/storage, and cellular signaling/communications^1,9^. Although many studies have shown that LLPS can play a vital role in normal physiological functions of cells^1,2,10,11^; it can be also associated with malfunctions^1,6,12,13^. Concentrations of protein increase several orders of magnitude inside the condensate^14–16^ compared to their endogenous levels. This often leads to toxic protein aggregation and nucleation of amyloid fibril formation associated with various human diseases, such as amyotrophic lateral sclerosis (ALS), Alzheimer’s disease (AD), and Parkinson’s disease (PD)^1,17–22^. It is widely accepted that intra- and inter-molecular interactions driving protein phase separation are embedded in the protein/peptide sequence and the respective structure^1,4,6,14,23^. In this context, the conformational properties of intrinsically disordered regions (IDRs), low complexity domains (LCDs), and prion-like domains (PLDs) are known to facilitate multivalent interactions that are prerequisites for condensate formation^23–28^. Further, the specific arrangement of amino acids in protein sequences under various conditions can regulate LLPS^23,27,29,30^ through common mechanisms that promote these multivalent interactions (such as electrostatic and cation-π interactions)^23,25,26,29,31^. The nature of the overall interactions between the proteins makes the condensates responsive to the cellular microenvironment^32,33^. By exploiting this knowledge, it is also possible to design artificial peptides/proteins with tunable LLPS properties^8,34,35^.

However, emerging evidence indicates that a significant proportion of proteins in the human proteome reside at concentrations just below their respective solubility limit^36^. The concentration levels not only depend on the extent of endogenous expression of individual proteins; but can also be greatly affected by the efficiency of the protein turnover machinery of the cell. The transition from soluble to LLPS state (reaching the saturation concentration) thus, is not associated with a very high energy barrier^2,33,37,38^. Seemingly, alterations such as post-translational modifications, changes in cellular or subcellular localization, the effect of counterions, and metabolites (such as ATP) can significantly modulate the phase behavior of various proteins^5,31,39–43^. Apart from intrinsically disordered proteins (IDPs), globular proteins (such as lysozyme^44^) are also capable of undergoing LLPS. Since the basis of most supramolecular assemblies (aggregates/precipitates/LLPS/crystals) is the intermolecular interactions, by tuning the extent of such interactions, it is experimentally feasible to explore conditions that drive LLPS of globular proteins as well.

Further, for any multivalent molecule with even the most-weakly attractive interactions, the null expectation is that there exists a concentration regime in which self-assembly will occur. While this prediction can be clearly confirmed by theory, simulation, and synthetic polymers, whether such a prediction holds true under experimentally accessible conditions for real proteins and polypeptides remain less well-established. Here, we assessed the ability of 23 different proteins/polypeptides with diverse structures/sequences to undergo homotypic phase separation in the presence and absence of the macromolecular crowder PEG-8000. Our results confirm that both folded and disordered proteins can be driven to form dynamic, reversible liquid-like condensates in a concentration-dependent manner. The driving forces and kinetics for assembly vary from protein to protein, and the observed saturation concentrations scale directly with the apparent intermolecular binding constant (K_D_). Moreover, our data confirm that a variety of distinct chemical modes can drive phase separation. To further explore these observations, we designed polypeptides based on neutral (Gly), hydrophobic (Val), positively (Arg), and negatively charged (Asp) amino acids and observed that even these simple model peptides could undergo pseudo-homotypic phase separation under appropriate solution conditions. Taken together, our results suggest that the observation that a protein or peptide can be driven to undergo phase separation under some solution conditions should be the null model for any in vitro system. Our results caution researchers in ascribing the functional significance to in vitro assays without consideration of the physiological relevance of those conditions. In parallel, our results also suggest that the regulation of intracellular phase transitions may be an unavoidable facet of cell biology, regardless of if the resulting assemblies are functional.

## Results

### Liquid-liquid phase separation (LLPS) of a diverse library of proteins

**Fig. 1:**
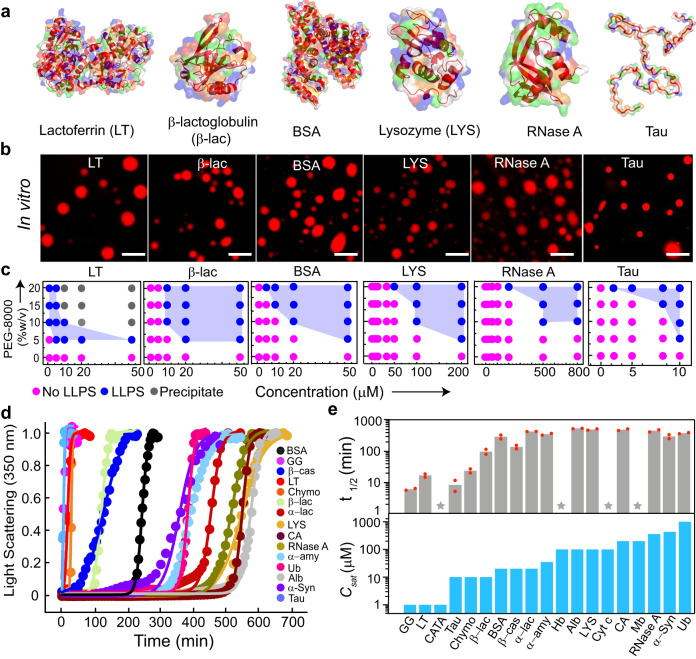
Liquid-liquid phase separation of various proteins in vitro. **a** Three-dimensional surface representation of selected proteins [LT (PDB ID: 1B0L), β-lac (PDB ID: 1QG5), BSA (PDB ID: 3V03), LYS (PDB ID: 1REX), RNase A (PDB ID: 1FS3), and Tau (PED00017e001)] with their embedded secondary structures (dark red). Positive, negative, and hydrophobic amino acids are represented in blue, red, and green colors, respectively. **b** Fluorescence microscopy images showing LLPS of selected NHS-Rhodamine labeled [10% (v/v) labeled to unlabeled] proteins (LT, β-lac, BSA, LYS, RNase A, and Tau) in the presence of 10% (w/v) PEG-8000. The samples were prepared in 20 mM sodium phosphate buffer (pH 7.4). Representative images are shown (*n* = *3*, independent experiments). The scale bar is 5 µm. **c** Schematic representation showing the phase regime of selected proteins (LT, β-lac, BSA, LYS, RNase A, and Tau) LLPS at varying protein and PEG-8000 concentrations. The different states are represented with various color codes. The pink color indicates no LLPS (soluble state), the blue color indicates LLPS (condensate state), and the grey color indicates precipitation. The experiment was performed three independent times with similar observations. **d** Static light scattering experiment (at 350 nm) showing the kinetics of protein condensate formation with time at their *C*_*sat*_ and in the presence of 10% (w/v) PEG-8000 (*n* = *2*, independent experiments). The light scattering values were normalized to 1. **e** ***Top panel***: t_1/2_ values depicting protein condensate kinetics determined from the sigmoidal fit from Fig. 1d. The data represent the mean for *n* = *2* independent experiments. ***Bottom panel***: *C*_*sat*_ of all proteins determined from the microscopic observation are represented with bar graphs. PEG-8000 was kept constant [10% (w/v)] to obtain a comparative measure of *C*_*sat*_ of all the proteins. The star symbol represents chromophore-containing proteins for which the light scattering experiment was not performed. The Y-axis values are in the log scale. *n* = *3* independent experiments. Source data are provided as a Source Data file.

To address if LLPS is a generic phenomenon of proteins, we first examined whether a diverse library of proteins could undergo LLPS in vitro in the presence of a molecular crowder (in our case, PEG-8000). We chose this library of proteins from multiple species with varied sequences, structures, and properties (Supplementary Table 1). Also, to exclude the influence of various cellular factors and other parameters such as salt, we have used a 20 mM sodium phosphate buffer (pH 7.4) in the presence of PEG-8000 as a molecular crowder. We first generated the three-dimensional surface image of proteins superimposed with their secondary structures using PYMOL (v 2.5.2) to understand the diversity of structures and distribution of charge (Fig. 1a and Supplementary Fig. 1). We then examined all the protein sequences in silico, using IUPred2A^45^, SMART^46^, CatGranule^47^ and PONDR^48^ for predicting the presence of IDRs, LCDs, their LLPS and disorder propensities, respectively. Our data revealed that a subset of proteins possesses LLPS propensity as well as sequence/s featuring intrinsic disorders/LCD regions. On the other hand, many proteins, such as lysozyme (LYS) and β-lactoglobulin (β-lac) did not exhibit any such features (Supplementary Fig. 2 and Supplementary Table 2). To test whether these proteins can undergo LLPS in vitro, we purified all the proteins using size exclusion chromatography (SEC) and examined for LLPS using fluorescence microscopy (by labeling the proteins with NHS-Rhodamine) in the presence of PEG-8000 (Fig. 1b and Supplementary Fig. 3). To construct the LLPS regimes, proteins at varying concentrations were incubated with different concentrations of PEG-8000 at physiological pH 7.4. We observed that all proteins undergo LLPS at different concentrations, thereby exhibiting a varied phase regime (Fig. 1c and Supplementary Fig. 4a) with different condensate size distributions (Supplementary Fig. 5). The integrity of all the proteins was evident from the protein band/s in SDS-PAGE after LLPS (Supplementary Fig. 6).

From this phase regime, we further determined the apparent saturation concentration (*C*_*sat*_) of the proteins in the presence of 10% (w/v) PEG-8000. Here, we define the apparent *C*_*sat*_ as the minimum protein concentration where we observed phase separation under the microscope within 12 h of waiting time. Note that the apparent saturation concentration is abbreviated as *C*_*sat*_ for easy referencing in the subsequent sections. Importantly, we do not know at present the exact saturation concentration for the proteins. However, we think that the *C*_*sat*_ determined using microscopy in our study is not significantly different from the saturation concentration of the proteins as we observe an increase in light scattering measurements (see later) along with microscopic observations.

Proteins such as lactoferrin (LT), γ-globulin (GG), and catalase (CATA) required as low as 1 µM concentration to undergo LLPS, while ubiquitin (Ub), α-Syn and RNase A required a very high protein concentration (≥500 µM) for phase separation (Supplementary Fig. 4a).

Further to support our microscopic observations, we calculated the dilute phase concentration after phase separation using the centrifugation method^49^ (Supplementary Fig. 7). For the proteins LT, β-cas, α-amy, LYS and RNase A, the *C*_*sat*_ determined from the microscopic study was approximately consistent with the *C*_*sat*_ calculated from the dilute phase protein concentration. Another subset of proteins (GG, CATA, Tau, Alb, Cyt c, Hb, CA, Mb, α-Syn, Ub) showed a slightly higher *C*_*sat*_ by microscopy than the *C*_*sat*_ estimated through centrifugation. This could be a case of overestimation due to the resolution limit of the grid of the microcopy-based phase regime. However, for Chymo, β-lac, BSA, and α-lac, the centrifugation estimate is higher than the microscopy estimate. This could possibly be due to the relatively smaller size of the condensates, for which the centrifugation speed used may not have been sufficient for the separation of dense and dilute phases^50^ (Supplementary Fig. 7).

To further evaluate the kinetics of LLPS, we performed a static light scattering experiment (at 350 nm) of each protein at their respective *C*_*sat*_ in the presence of 10% (w/v) PEG-8000 over time (Fig. 1d). Similar to their phase behavior, the data revealed that kinetics of LLPS also varied across different proteins (Fig. 1d, e). At the end of the light scattering experiments, the condensate formation by proteins was further verified using differential interference contrast (DIC) microscopy (Supplementary Fig. 4b). The light scattering data were fitted with a sigmoidal growth kinetics model (see method section) and the t_1/2_ for LLPS was determined for all the proteins. The data revealed that many proteins with low *C*_*sat*_ exhibited faster LLPS kinetics in contrast to proteins with higher *C*_*sat*_ (Fig. 1e). Overall, the data provides promising evidence in support of LLPS being a generic phenomenon of proteins at crowded microenvironment.

### Role of PEG in protein phase separation

**Fig. 2:**
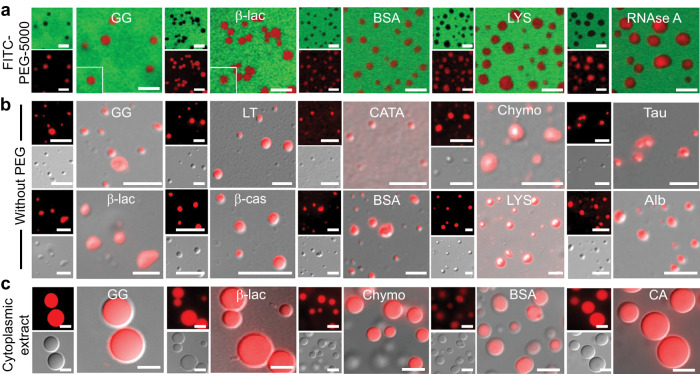
Role of PEG in liquid-liquid phase separation of proteins in vitro. **a** Representative confocal microscopy images of selected NHS-Rhodamine labeled proteins [10% (v/v) labeled to unlabeled] (GG, β-lac, BSA, LYS and RNase A) in the presence of 10% PEG (w/v) (5% FITC-labeled PEG-5000 + 5% PEG-8000) showing LLPS with no PEG sequestration inside the condensates. All the samples were prepared under identical conditions using 20 mM sodium phosphate buffer (pH 7.4) and FITC-labeled PEG-5000 was added before the proteins undergo LLPS. The scale bar is 5 µm. **b** Representative fluorescence, DIC microscopy and DIC/fluorescence merged images of the selected NHS-Rhodamine labeled protein [1:10 (v/v) labeled to unlabeled protein] condensates at different conditions (Supplementary Table 3) in the absence of PEG-8000. Note the liquid condensate formation in the absence of PEG-8000 by other proteins is shown in Supplementary Fig. 8d. The scale bar is 5 μm. **c** Representative fluorescence microscopy images showing LLPS in cytoplasmic extract for selected NHS-Rhodamine labeled proteins (GG, β-lac, Chymo, BSA and CA). The scale bar is 5 μm. All the experiments were performed three independent times with similar observations.

PEG is a non-ionic polymer that increases the tendency of the protein to self-assemble into condensates by inducing intermolecular interaction^51^. Previous studies indicated that PEG might not be considered as an inert crowder as it might interact with proteins where some amino acids have been shown to possess higher interaction potential with PEG^52^. To examine the possibility of whether PEG is directly participating in the liquid condensate formation in our studies and in the present experimental condition, we performed phase separation experiments with a subset of protein in the presence of FITC-labeled PEG (5% FITC-labeled PEG-5000 + 5% PEG-8000). We observed that there is no selective sequestration of PEG inside the protein condensates when added both before or after the condensate formation as evident from the confocal microscopy images (Fig. 2a and Supplementary Fig. 8a, b). Since the fluorescence intensity is linearly proportional to concentration, we calculated the apparent partition coefficient using the fluorescence intensity of PEG inside (${PE}{G}_{{inside}}$) and outside $({PE}{G}_{{outside}})$ of the condensate. For instance, the apparent partition coefficient in the case of β-lac is 0.01 = ~0. Indeed, this was the case for the subset of proteins we tested, confirming that there is no PEG sequestration inside the protein condensates under study. This indicates that condensate formation is majorly driven by protein-protein interactions.

Further, the effect of PEG polymer length in protein LLPS was evaluated using 10% (v/v) PEG-300. We studied the condensate formation using 10% (v/v) PEG-300, however, at the same *C*_*sat*_ of proteins. We observed the condensate formation of proteins but with slower kinetics in comparison to the proteins in the presence of PEG-8000. A subset of proteins such as GG, Chymo, β-lac and BSA exhibited LLPS after 24 h, while LYS, Alb, RNase A and Ub required 48 h for observable condensate formation (Supplementary Fig. 8c). This suggests that lowering the molecular weight of PEG might increase the time required for the condensate formation of protein, however, it does not alter the propensity of protein phase separation in identical buffer conditions.

More importantly, we also examined the condition where the proteins can phase separate readily in the absence of PEG. For this, we performed the LLPS study using purified proteins from size-exclusion chromatography and tested different conditions (high protein concentration, pH and/or NaCl) for phase separation in the absence of PEG-8000 (Fig. 2b, Supplementary Fig. 8d and Supplementary Table 3). We observed that GG and LT undergo LLPS at high protein concentrations in the absence of PEG. For some proteins, a change in pH (LYS and RNase A) or the addition of salt (BSA, Alb and β-cas) induced the condensate formation. However, in the case of α-Syn, both alteration of pH (pH 5.5) and addition of salt (1 M) was required to induce condensate formation at a very low protein concentration (10 μM) in contrast to the high concentration (600 μM) required for α-Syn to undergo LLPS in presence of PEG-8000. Hence, our study indicates that proteins can undergo phase separation in the absence of PEG (crowder), but under different conditions. Therefore, molecular crowder such as PEG might only facilitate intermolecular interaction via depletion mechanism and/or osmotic pressure effect^53^, however, protein-protein interactions play a major role in condensate formation.

In the end, we performed LLPS reactions of all proteins using cytoplasmic extract^54^ of HeLa cells to access the effect of other biomolecules on protein LLPS under physiological crowding conditions (Fig. 2c and Supplementary Fig. 9a, b). We used NHS-Rhodamine labeled proteins at their respective *C*_*sat*_ for LLPS in the cellular extract. Interestingly, all proteins (except Ub and α-Syn) showed LLPS in cell extract, however, with much larger condensate size compared to their corresponding condensate in the PEG-buffer system (Fig. 2c and Supplementary Fig. 9b). In the case of α-Syn, we observed the aggregate formation and Ub showed no LLPS even after a long incubation suggesting more specific conditions might be required for the protein condensate formation in cell lysate (Supplementary Fig. 9b). The condensate fusions upon contact and FRAP study by selected proteins revealed that all these protein condensates in cytoplasmic extract possess liquid-like behavior (Supplementary Fig. 9c–e).

### Liquid-like property of the phase separated condensates

**Fig. 3:**
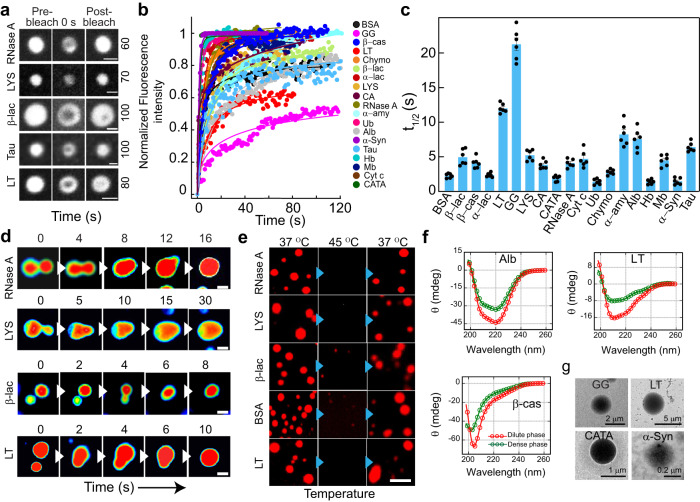
Liquid-like properties of the various protein condensates. **a** Representative images showing the liquid condensates (immediately after formation, 0 h) during FRAP [before bleaching, at bleaching (0 s), and after bleaching (respective recovery time shown at the right side in second)] for selected proteins (RNase A, LYS, β-lac, Tau and LT). The scale bar is 2 μm. The images are represented in the ‘grey’ lookup table (LUT) for better visualization. **b** Normalized FRAP curves (in arbitrary units) obtained for all the protein condensates at 0 h (immediately after LLPS) are plotted against time. *n* = *3* independent experiments were performed. **c** The bar plot of t_1/2_ values showing fluorescence recovery after photobleaching of protein condensates at 0 h. The data represent the mean ± s.e.m. for *n* = *3* independent experiments from multiple condensates. **d** Time-lapse images showing fusion events of condensates formed by selected proteins over time (RNase A, LYS, β-lac, and LT). Images are represented in ‘royal’ LUT for better visualization. Representative results are shown. The scale bar is 5 μm. The experiment was repeated two times. **e** Fluorescence microscopy images showing thermo-reversibility (37 °C → 45 °C → 37 °C) of selected NHS-Rhodamine labeled [10% (v/v)] protein condensates formed at their respective *C*_*sat*_ in the presence of 10% (w/v) PEG-8000 (at 0 h). Representative images are shown. The experiment was performed two times with similar observations. The scale bar is 5 μm. **f** CD spectroscopic analysis of selected proteins (Alb, β-cas, and LT) showing no substantial changes in the secondary structural conformation of the dense and dilute phase of the proteins upon phase separation. The red and green colors indicate protein CD spectra of dilute and dense phases in the presence of PEG-8000 [10% (w/v)], respectively. *n* = *2*, independent experiments were performed. **g** Representative TEM images showing the morphology of protein condensates formed by GG, LT, CATA, and α-Syn. *n* = *2* independent experiments were performed. Source data are provided as a Source Data file.

Typical characteristics of phase separated condensates include condensate fusion upon contact, temperature reversibility, and rapid fluorescence recovery after photobleaching (FRAP). To examine the dynamic nature of the molecules inside the condensates, we performed FRAP using 10% (v/v) NHS-Rhodamine labeled proteins. At the initial time of condensate formation (0 h), most of the proteins showed rapid recovery of fluorescence (~80-100% recovery) with a short half-life (t_1/2_) (< 5 s); while a few proteins showed partial recovery (e.g., LT and GG showed 50-60% recovery) with higher t_1/2_ values (>10 s) (Fig. 3a–c and Supplementary Fig. 10a). We hypothesized that extensive intermolecular interactions might result in the viscoelastic transition leading to reduced fluorescence recovery (also supported by their very low *C*_*sat*_). The liquid-like property of the condensates was further supported by fusion events (Fig. 3d, Supplementary Fig. 10b and Supplementary movie 1, 2) and the dissolution of condensates upon increased temperature (at 45 °C). The protein condensates, however, reappeared upon incubating back to 37 °C (Fig. 3e and Supplementary Fig. 10c), suggesting their thermo-reversible property. To examine whether LLPS is associated with the conformational transition of the proteins, we isolated the dense and dilute phases of all proteins through centrifugation and performed circular dichroism (CD). We observed no substantial change in secondary structure/s upon phase separation (Fig. 3f and Supplementary Fig. 11) as CD spectra of proteins in dense and dilute phases are essentially similar. Note, we performed CD spectroscopy of dense phase proteins after dilution. This is unavoidable for CD study due to very high dynode voltage and light scattering of the original dense phase suspension. Therefore, we further performed FTIR spectroscopy to analyze the secondary structure of the intact dilute and dense phase of the proteins. Our deconvoluted FTIR spectra (Supplementary Fig. 12) and secondary structure estimation data (Supplementary Table 4) revealed that the gross secondary structure remains the same after phase separation for all the proteins with some subtle secondary structural changes for a few proteins. The morphology of liquid condensate by various protein LLPS samples were further examined using transmission electron microscopy (TEM). The TEM micrographs mostly showed circular protein-rich condensates (Fig. 3g and Supplementary Fig. 10d). The data, therefore, suggest that proteins can form thermo-reversible, liquid condensates without significant alteration in their secondary structures.

### Maturation and rigidification of protein condensate over time

**Fig. 4:**
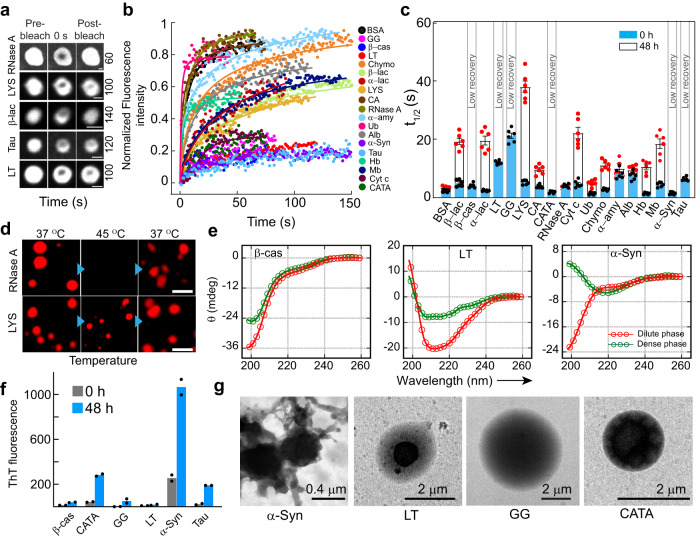
The viscoelastic transition of protein condensates. **a** Representative images showing selected protein condensates after LLPS (48 h) during FRAP analysis [(before bleaching, at bleaching (0 s), and after bleaching (respective recovery time shown at the right side in second)]. The images are represented in ‘grey’ LUT for better visualization. RNase A shows complete fluorescence recovery, whereas, LYS, β-lac, Tau and LT show partial recovery. The scale bar is 2 μm. **b** Normalized FRAP (in arbitrary units) profile of the phase separated condensates at 48 h by various proteins showing different fluorescence recovery. A subset of protein condensates after 48 h show reduced fluorescence recovery, indicating that they might undergo rigidification over time. *n* = *3* independent experiments were performed. **c** The t_1/2_ values were calculated from FRAP of all proteins at 0 h (blue) and 48 h (white), showing slow fluorescence recovery of protein condensates after 48 h. Notably, t_1/2_ values could not be calculated for β-cas, LT, GG, CATA, α-Syn and Tau due to the negligible recovery after photobleaching. The data represent the mean ± s.e.m. for *n* = *3* independent experiments. **d** Fluorescence microscopy image of NHS-Rhodamine labeled condensates (10% v/v) by RNase A (48 h) showing thermo-reversibility upon heating (45 °C) and cooling (37 °C). The LYS condensates did not dissolve upon heating, suggesting a viscoelastic transition after 48 h. The scale bar is 5 μm. Representative images are shown and the experiment was performed two times with similar observations. **e** CD spectra of selected proteins (β-cas, LT and α-Syn) demonstrating the secondary structure of the dilute (red) and dense (green) phases of proteins at 48 h (*n* = *2*, independent experiments). **f** ThT fluorescence intensity (in arbitrary units) of different proteins immediately after LLPS (0 h) and after 48 h are shown. An increase in ThT intensity for α-Syn at 48 h suggests the formation of ThT positive, amyloid aggregates. The data represent the mean for *n* = *2* independent experiments. **g** TEM micrographs showing the appearance of multiphasic architectures by various protein condensates (α-Syn, LT, GG and CATA), while amyloid fibril formation by α-Syn condensate. *n* = *2* independent experiments were performed. Source data are provided as a Source Data file.

The viscoelastic transition of protein condensates is often associated with toxic amyloid fibril formation in various neurodegenerative diseases such as ALS, AD, and PD^17–20^. However, such viscoelastic transition can also help in various cellular functions^5,6,55^ including oocyte dormancy (Balbiani body^56^) and heterochromatin assembly^57–59^. We wanted to investigate whether the condensates formed by the various proteins in our study also undergo rigidification with time. We incubated various protein condensates for 48 h (at 37 °C) and performed FRAP and temperature reversibility (Fig. 4a–d and Supplementary Fig. 13a, b) studies. FRAP analysis of condensates at 48 h revealed substantially slower recovery (higher t_1/2_) for most of the proteins compared to freshly formed liquid condensate (0 h) (Fig. 4c). Intriguingly, a few proteins (GG, LT, Tau, α-Syn, β-cas, and CATA) did not recover after photo-bleaching at 48 h (Fig. 4c), suggesting their viscoelastic transition, which might be due to the change in material property because of the extent of intermolecular interaction and protein arrangement inside the condensate. This was also consistent with the thermo-reversibility study as these condensates did not dissolve upon increasing the temperature to 45 °C (Fig. 4d and Supplementary Fig. 13b). To examine the possible structural changes due to rigidification, we performed CD spectroscopy for a subset of proteins (which showed negligible fluorescence recovery after 48 h) (Fig. 4e and Supplementary Fig. 14). The data suggest that except for α-Syn, the rest of the proteins did not undergo substantial structural changes during the viscoelastic transition. The FTIR spectroscopic study of the dense and dilute phase of these proteins further showed that except α-Syn, no other proteins showed significant structural changes upon phase separation and subsequently their viscoelastic transition after 48 h (Supplementary Fig. 15), consistent with CD data. To examine whether the loss of dynamicity by any of the proteins was associated with amyloid fibril formation, we performed ThT (which binds to amyloid aggregates) fluorescence assay^18^. The data suggest that except α-Syn (bind strongly with ThT as expected^18^), no other proteins showed any significant ThT binding (Fig. 4f). This indicates that either crystal-like native packing/protein vitrification and/or amorphous aggregation might result in their rigidification^1,2,6,22,60^. To further characterize the morphology of the condensates after 48 h, we analyzed the condensates using TEM (Fig. 4g and Supplementary Fig. 13c). The TEM images of GG and LT condensates showed a multiphasic nature as evident from different electron-dense/sparse regions, indicating protein assembly in the condensate (Fig. 4g). We also observed aggregate-like morphology around α-Syn condensates as previously reported^18,40,61,62^ (Fig. 4g). The data suggest that partial or full rigidification might occur for protein condensates upon ageing with or without structural transition.

### Correlation of sequence and structure specific parameters with *C*_*sat*_ for various proteins LLPS

**Fig. 5:**
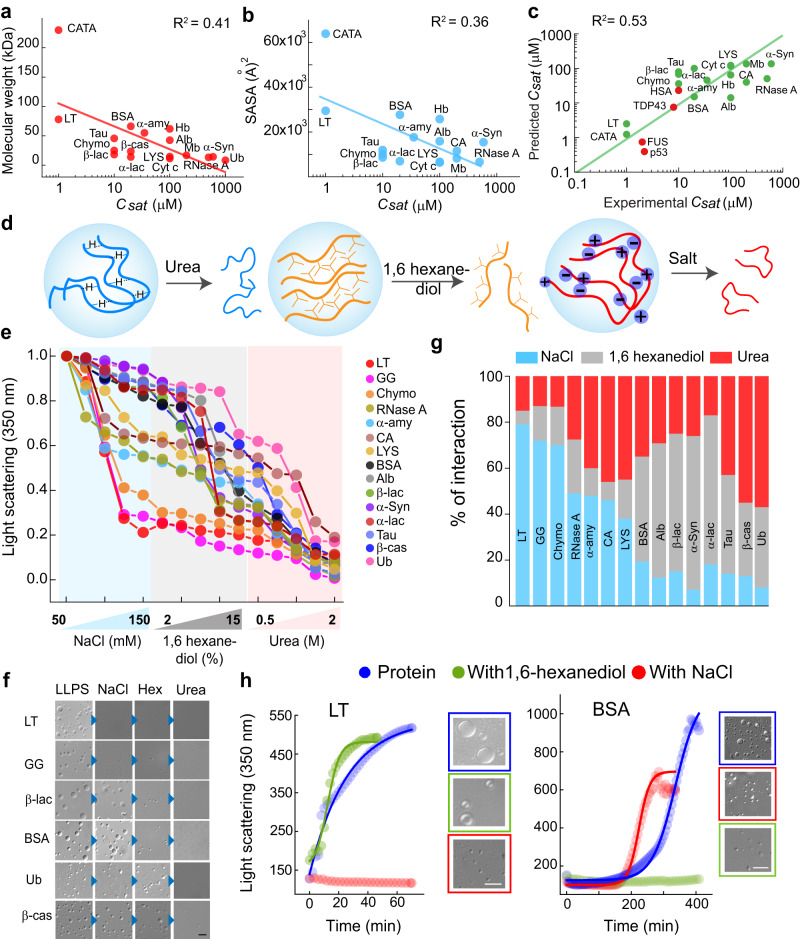
Intermolecular interactions govern LLPS of all proteins. Correlation plots between the (**a**) molecular weight of proteins and their respective *C*_*sat*_ (**b**) SASA and *C*_*sat*_ of proteins, showing negative correlation (in semi-log scale). **c** Correlation plot (Pearson’s correlation coefficient = 0.77) of predicted *C*_*sat*_ and experimental *C*_*sat*_ showing a positive correlation (log-log scale) for most of the proteins. The green color indicates proteins included in our study and the red color indicates proteins not included in our study for LLPS and were tested using this predictive model. The structure of FUS (AlphaFold ID: AF-P35637-F1) and TDP43 (AlphaFold ID: AF-Q13148-F1) were taken from the AlphaFold database, whereas the structural information of p53 (PDB ID: 8F2H) and HSA (PDB ID: 4LB2) was obtained from the Protein data bank. Notably, the *C*_*sat*_ values could not be calculated using the predictive model for GG, β-cas and, Ub since the information regarding the structural conformation were not available in the Protein data bank. **d** Schematic representation showing additives, which disrupt the different intermolecular interactions (salt, electrostatic interaction; 1,6 hexanediol, hydrophobic interaction; and urea, hydrogen bonding). **e** Normalized static light scattering at 350 nm showing a decrease in light scattering value by the titration of different concentrations of additives on preformed protein condensates. **f** Representative DIC images confirming the absence or presence of condensates in the presence of additives (150 mM NaCl, 15% (w/v) 1,6 hexanediol and 2 M urea) during the light scattering experiment. The scale bar is 5 µm. *n* = *2*, independent experiments were performed. **g** Stacked columns showing the relative percentage of different intermolecular interactions responsible for each protein LLPS. Blue, light grey and red color represent the percentage of electrostatic, hydrophobic and hydrogen bonding interactions, respectively. **h** The light scattering measurement at 350 nm showing relative inhibition of LLPS (LT and BSA) in the presence of either 150 mM NaCl (red) or 10% (w/v) 1, 6-hexanediol (green). All the LLPS experiments were performed in the presence of 10% (w/v) PEG-8000. The blue color indicates light scattering measurement of protein in the presence of 10% (w/v) PEG-8000 only. Representative DIC images showing the absence or presence of condensate formation in light scattering experiments. The scale bar is 5 µm, *n* = *2*, independent experiments were performed. Source data are provided as a Source Data file.

We hypothesized that proteins undergo LLPS through different intermolecular interactions based on their surface-exposed charge, hydrophobicity and through H-bonding. This is due to the different structural fold/s and amino acid sequences of the proteins. According to the Flory Huggins (FH) theory^63^, the important criteria driving phase separation are (a) the length of residues capable of intermolecular interactions [which is directly proportional to molecular weight (MW)] and (b) their respective interaction strengths. However, this is true for a (semi)-flexible polymer, but for globular protein exposed residues scale with the surface area. In the limit of a spherical globule, radius of gyration, ${Rg}=B{N}^{0.33}$ [where N is the number of residues and B is a constant], while the surface area (SA) of a sphere is defined as ${SA}=4\pi {\left({Rg}\right)}^{2}$, such that ${SA}=4\pi {(B{N}^{0.33})}^{2}$ → SA is proportional to ${N}^{\frac{2}{3}}$ (or, given all amino acids are approximately the same mass, SA is proportional to $M{W}^{\frac{2}{3}}$). We plotted the molecular weight and also (MW)^0.66^ of all proteins with the respective *C*_*sat*_. However, we do not observe any apparent correlation between the parameters (Fig. 5a and Supplementary Fig. 16a). In both cases, we see an overall negative correlation and proteins having a similar range of molecular weight do show very different *C*_*sat*_. Although, achieving a perfect correlation between the sequence-specific quantities and *C*_*sat*_ is unlikely as the *C*_*sat*_ depends on many factors and molecular weight is just one of them. Further, the FH theory best explains the liquid-liquid phase separation of homopolymers and does not account for the complexity of protein including sequence variations and electrostatic interactions^64,65^. Thus, the protein LLPS might not be explained using FH theory by interaction strength alone. Various factors, such as protein conformation and its susceptibility to change with concentration, length of the protein, sequence specificity, etc. might also dictate protein LLPS.

We further derived the solvent-accessible surface area (SASA) using available PDB structure files for the proteins. For intrinsically disordered proteins, we used a solved structure ensemble from Protein Ensemble Database (proteinensemble.org) and calculated the averaged properties from the structures. We calculated SASA using an existing SASA algorithm present in the VMD (Visual Molecular Dynamics)^66^ software package. We then plotted the SASA values and the *C*_*sat*_ for all the proteins (Fig. 5b) and observed that the correlations are weak, suggesting that multiple factors affect the determination of *C*_*sat*_. Further, using the solvent-accessible residues, we decomposed each protein into polar, hydrophobic, aromatic, and charge residues contributions and calculated global weighting parameters, which enable a 4-parameter model to be globally fit to the data i.e. an equation of the format

1 $${C}_{{sat}}=A1\times {N}_{{Polar}}+A2\times {N}_{{Hydrophobic}}+A3\times {N}_{{Charge}}+A4\times {N}_{{Aromatic}}$$

where *C*_*sat*_ is measured, N_* are calculable from sequence and A1-A4 are constants (unit concentration) that can be globally fit. We indeed find that the best-fit parameters led to the relation that predicts the *C*_*sat*_:

2 $${\log }_{10}\left({C}_{{sat}}\right)=	 -0.041\times {N}_{{Polar}}+0.016\times {N}_{{Hydrophobic}}\\ 	 -0.005\times {N}_{{Charge}}+0.026\times {N}_{{Aromatic}}+2.24$$

Here, each quantity (${N}_{{Polar}}$, ${N}_{{Hydrophobic}}$, ${N}_{{Charge}}$, and ${N}_{{Aromatic}}$) is defined as the effective exposure of those respective residues on the surface. By supplying each quantity ${N}_{{Polar}}$, ${N}_{{Hydrophobic}}$, ${N}_{{Charge}}$, and ${N}_{{Aromatic}}$ from the structure, one can predict the *C*_*sat*_ using the above equation. Note, we used log (*C*_*sat*_), as the energy is proportional to the log of concentration. We compared the predicted *C*_*sat*_ to the experimentally determined *C*_*sat*_ (Fig. 5c). Further, we also validated this predictive model of the equation using a few other proteins (FUS, TDP43, p53 and HSA), which are not included in our study for LLPS. Important to note that the structure of FUS (AlphaFold ID: AF-P35637-F1) and TDP43 (AlphaFold ID: AF-Q13148-F1) was taken from the AlphaFold database; whereas the structural information of p53 (PDB ID: 8F2H) and HSA (PDB ID: 4LB2) was acquired from the Protein data bank. Interestingly, we observed that the *C*_*sat*_ predicted using the model approximately correlated with the experimental *C*_*sat*_ as reported in the literature^31,67–69^. The correlation plot shows that there is an overall positive correlation with the Pearson correlation coefficient of 0.77. However, the correlation is not perfect. We further tried different sequence-specific parameters and observable properties of liquid condensate with *C*_*sat*_, however, we did not find any apparent correlation (Supplementary Fig. 16a–h). This might be due to the fact that the driving forces of phase separation are complex and diverse, which could not be easily extrapolated at present.

### Role of various intermolecular interactions responsible for protein LLPS

To investigate the role of various inter-molecular interactions (electrostatic, H-bonding and hydrophobic) responsible for protein LLPS, we performed condensate dissolution assay using a sequential titration of NaCl (disrupts electrostatic interaction)^13,32^, 1,6 hexanediol (disrupts hydrophobic interaction)^70,71^ and urea (disrupts H-bonding and van der Waals interaction)^72,73^ (Fig. 5d). We used up to 2 M urea, as this concentration range might not significantly unfold globular proteins^74,75^ (Supplementary Fig. 17), rather may break the inter-molecular H-bonding for condensate dissolution. We further hypothesized that the addition of one or a combination of these molecules will disrupt the preformed condensate and thereby reveal the nature of intermolecular interaction responsible for its formation/stabilization. The condensate dissolution was assayed using static light scattering at 350 nm (Fig. 5e) for each protein condensate immediately after its formation. The dissolution of the preformed condensates was further verified using DIC imaging of the LLPS solution at the highest concentration of each additive (Fig. 5f and Supplementary Fig. 18a). The data showed that the preformed condensates of LT and GG were mostly disrupted by the addition of salt ( > 70% decrease in light scattering value). While considerably lower effect was observed on the addition of 1,6 hexanediol and urea in these proteins (Fig. 5g). This suggests that electrostatic interaction is playing a major role in phase separation (or maintaining the phase separated state) of LT and GG. In contrast, the light scattering value of BSA, Alb, α−Syn and β−lac condensates mostly dropped by the addition of 1,6 hexanediol suggesting that hydrophobic interaction played a major role in the formation and/or maintaining these protein condensates. Indeed, the ANS binding study (probing the exposed hydrophobic surface^76^) showed an increase in ANS fluorescence for BSA and a moderate increase for Alb, CATA and β−lac, suggesting hydrophobic interactions might play a role in LLPS of these proteins (Supplementary Fig. 18b). On the other hand, urea showed a major impact on the dissolution of Ub and β−cas condensates, indicating that these proteins undergo phase separation majorly by H-bonding and other van der Waals interactions (Fig. 5g). Important to note that in the titration experiments, the second additive and the third additive is not purely in phosphate buffer but in the presence of the previous additive. To rule out the possibility that the sequence of additives in titration experiments might affect the relative contribution of each interaction for LLPS, we performed a condensate dissolution assay using the altered sequence of additives for selected proteins. The data suggest that the sequence of addition does not alter the outcome of intermolecular interactions responsible for condensate formation by dissolution assay (Supplementary Fig. 18c). Further, based on the decrease in light scattering values, we calculated the relative percentage of three major types of interactions responsible for individual protein phase separation. Our data clearly suggest that either or combination of electrostatic, H-bonding and hydrophobic interactions are responsible for condensate formation and stabilization (Fig. 5g). However, the mode/extent of intermolecular interactions may differ depending upon the microenvironment and post-translational modifications of the protein^31^. For instance, in the case of α−Syn, the phosphomimetic mutation, S129E undergoes phase separation faster with a lower *C*_*sat*_ (200 μM) in comparison to the wild-type (Supplementary Fig. 19a). Also, upon titrating S129E and wild-type protein with different additives, the nature of intermolecular interaction driving phase separation differs (Supplementary Fig. 19b).

After predicting that proteins might mostly use either electrostatic or hydrophobic (or in combination) interactions for LLPS, we examined the kinetics of LLPS for selected proteins in the presence of salt, NaCl or 1,6-hexanediol using static light scattering (at 350 nm) (Fig. 5h and Supplementary Fig. 18d). Our data showed that LLPS of LT and Chymo (as predicted electrostatic interaction for LLPS) was largely inhibited by the addition of 150 mM NaCl; while there was no effect due to the presence of 10% (w/v) 1,6-hexanediol. In contrast, LLPS of BSA and Alb (with ANS binding due to exposed hydrophobic surface) was substantially inhibited by the presence of 10% (w/v) 1,6-hexanediol, but no difference in scattering intensity was observed in the presence of 150 mM NaCl (Fig. 5h and Supplementary Fig. 18d).

### Protein-protein interaction strength determines *C*_*sat*_

**Fig. 6:**
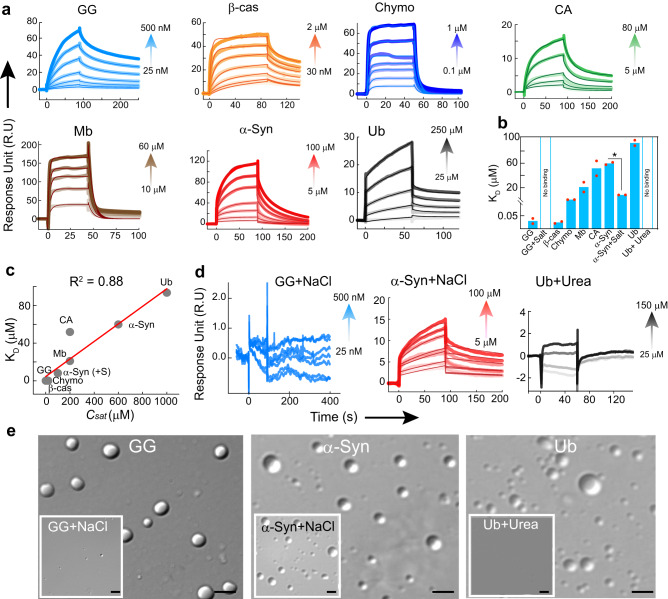
Correlation of homotypic protein-protein interaction and phase separation by proteins. **a** SPR sensogram of GG, Chymo and β-cas showing strong binding at low concentrations resulting in the low K_D_. CA and Mb showing moderate binding while α-Syn and Ub exhibits weak binding. *n* = *2*, independent experiments were performed. **b** A bar graph representing K_D_ of proteins. The data represent the mean for *n* = *2* independent experiments. The statistical significance was calculated using a two-tailed t-test (95% confidence interval) with no adjustments (*p* values, *p* < 0.001, *p* < 0.002, *p* < 0.033, and *p* > 0.12 indicated by (***), (**), (*) and (ns), respectively). The *p* value of α−Syn Vs α−Syn+salt is 0.023. **c** A correlation plot (R^2^ value: 0.88) between K_D_ of proteins and their respective *C*_*sat*_ showing a linear correlation. **d** SPR sensogram of GG and Ub showing drastic inhibition of the intermolecular interaction, in the presence of 150 mM salt and 2 M Urea, respectively. In contrast, α-Syn show a significant increase in inter-protein interaction (low K_D_) in the presence of 150 mM salt. *n* = *2*, independent experiments were performed. **e** Representative DIC microscopy images of GG and α-Syn confirming the presence/absence of condensates in the presence/absence of NaCl. Ub showing no condensate formation in the presence of urea as compared to control (without urea). The experiment was performed three independent times with similar observations. The scale bar is 5 µm. Source data are provided as a Source Data file.

To find the correlation between intermolecular interaction strength and saturation concentration required for protein LLPS (*C*_*sat*_), we performed the homotypic protein-protein interaction using a label-free technique of surface plasmon resonance (SPR) spectroscopy. We selected a subset of proteins based on their *C*_*sat*_ (low, intermediate and high) and immobilized the protein on the CM3/CM5 chips. The different concentrations of respective proteins were allowed to pass through the microfluidic channel, enabling interaction with the immobilized protein. Using one state/two state model, we fitted the resultant response curves and the respective K_D_ (binding affinity) values were determined. Our results showed strong protein-protein interaction for GG, Chymo and β−cas (low K_D,_ < 500 nM). Whereas Mb and CA showed an intermediate tendency for homotypic protein-protein interaction and a much low interaction tendency was showed by α−Syn and Ub (high K_D_, >60 μM) (Figs. 6a and b). When we plotted the correlation between K_D_ and respective *C*_*sat*_, we found a strong correlation between the binding affinity of proteins and their respective *C*_*sat*_ (Fig. 6c). This suggests that binding affinity/interaction strength determines the tendency and/or saturation concentration required for LLPS^77^. To further delineate how inter-protein interaction strength dictates the *C*_*sat*_, we performed SPR study of GG, α-Syn in the presence of 150 mM NaCl and Ub in the presence of 2 M Urea. In the presence of NaCl, GG showed no effective intermolecular interaction (Fig. 6d), which is consistent with the fact that LLPS of GG indeed occurs through electrostatic interaction and is inhibited in the presence of salt. Interestingly our previous study showed that in the presence of salt, an increase in the tendency of α-Syn phase separation occurs with a drastic reduction in *C*_*sat*_^40^. We found that the K_D_ of α-Syn in the presence of NaCl is ~7 fold lower as compared to α-Syn, signifying strong binding, which is also consistent with their respective *C*_*sat*._ Furthermore, to understand the importance of H-bonding for LLPS, we chose Ub for the determination of interaction strength in the presence and absence of 2 M urea. We indeed found no intermolecular binding affinity (accurate K_D_ could not be determined due to a very low response unit) of Ub in the presence of urea as compared to the control (without urea) (Fig. 6d, e). The data suggest that the strength of intermolecular interaction dictates the tendency, feasibility and saturation concentration required for protein LLPS. This might be tightly regulated in the cellular milieu to promote or prevent the protein LLPS as per need.

### Minimalistic peptide-based model determining different intermolecular interactions responsible for phase separation

**Fig. 7:**
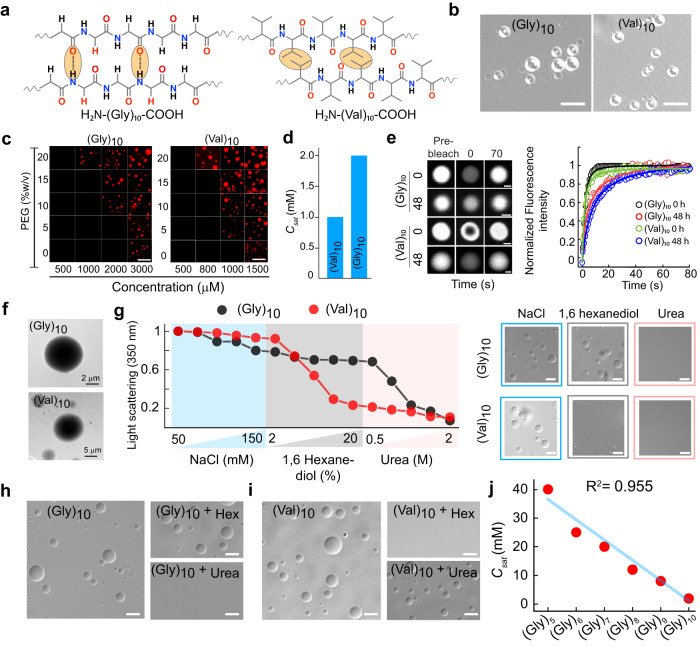
Liquid-liquid phase separation of neutral homo polypeptides. **a** Schematic representation showing the different possible modes of intermolecular interactions by homo-polypeptides by Gly and Val for their LLPS. **b** Representative DIC microscopy images showing condensate formation by (Gly)_10_ and (Val)_10_ at 2 mM and 1 mM concentration, respectively in the presence of 10% (w/v) PEG-8000. The scale bar is 5 µm. The experiment was performed three independent times with similar observations. **c** The phase regime of NHS-Rhodamine labeled polypeptides [1:10 (v/v) of labeled versus unlabeled peptide] at varying PEG-8000 and polypeptide concentrations in 20 mM sodium phosphate buffer at pH 7.4. The scale bar is 5 μm. The experiment was performed three independent times with similar observations. **d** Bar graph representing *C*_*sat*_ of (Gly)_10_ and (Val)_10_ in the presence of 10% (w/v) PEG-8000. **e** ***Left:*** Representative FRAP images (before bleaching, at bleaching, and after bleaching) of (Gly)_10_ and (Val)_10_ condensates at 0 h and 48 h (represented in ‘grey’ LUT). The scale bar is 2 μm. ***Right:*** Normalized FRAP (in arbitrary units) profile showing complete fluorescence recovery of the phase separated condensates (at 0 h and 48 h) of polypeptides (Gly)_10_, and (Val)_10_ (*n* = *3*, independent experiments). **f** Representative TEM images of (Gly)_10_ and (Val)_10_ condensates formed immediately after LLPS (0 h). *n* = *2*, independent experiments were performed. **g** ***Left panel*:** Static light scattering measurements of polypeptides at 350 nm showing the effect of increasing concentration of various additives at LLPS condition. To carry out the experiment, the LLPS mixture of (Gly)_10_ and (Val)_10_ at *C*_*sat*_ in the presence of 10% (w/v) PEG-8000 are treated with increasing concentration of NaCl (50–150 mM), followed by 1,6 hexanediol (2–20% w/v) and urea (0.5–2 M), respectively. ***Right panel:*** Representative DIC images confirming the absence or presence of condensates in the presence of additives during the light scattering experiment. The scale bar is 5 µm. The experiment was repeated twice with similar observations. **h**, **i** Representative DIC images of (Gly)_10_ and (Val)_10_ showing the effect of 1,6 hexanediol and urea for the phase separation. Respective polypeptides at *C*_*sat*_ are used as control. The scale bar is 5 µm. The experiment was repeated two times with similar observations. **(j)** A correlation plot of *C*_*sat*_ and length of (Gly)_n_ polypeptides (R^2^ value: 0.955) suggests that with an increase in the Gly polypeptide length, the *C*_*sat*_ decreases linearly. The experiment was repeated twice with similar observations. Source data are provided as a Source Data file.

**Fig. 8:**
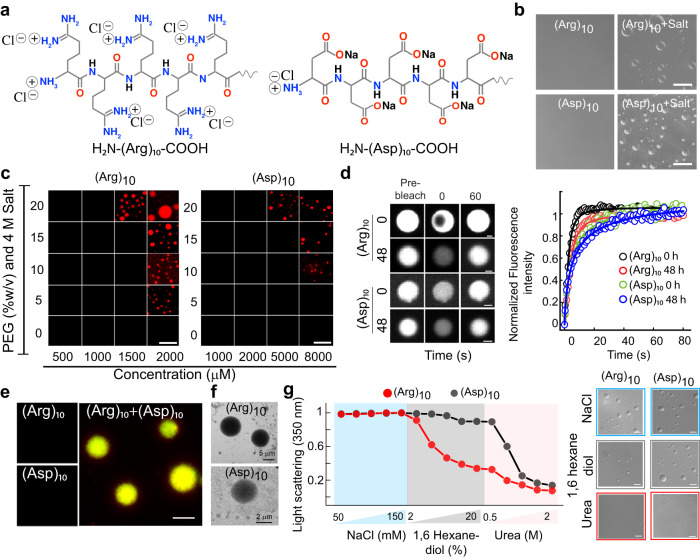
Liquid-liquid phase separation of charged polypeptides. **a** Schematic representation showing charge and side chain of Arg and Asp polypeptides. **b** Representative DIC microscopy showing liquid condensate formation of (Arg)_10_ and (Asp)_10_ condensates at 2 mM and 8 mM concentrations, respectively in the presence of 4 M salt and 10% (w/v) PEG-8000. The scale bar is 5 µm. Polypeptides at *C*_*sat*_ in the absence of salt were used as controls. The experiment was performed three independent times with similar observations. **c** The phase regime of NHS-Rhodamine labeled polypeptides [1:10 (v/v) of labeled versus unlabeled peptide] at varying PEG-8000 and polypeptide concentrations in 20 mM sodium phosphate buffer, in the presence of 4 M salt at pH 7.4. The scale bar is 5 μm. The experiment was performed three independent times with similar observations. **d** ***Left panel:*** Representative FRAP images (before bleaching, at bleaching, and after bleaching) of (Arg)_10_ and (Asp)_10_ condensates at 0 h and 48 h (represented in ‘grey’ LUT). The scale bar is 2 μm. ***Right panel:*** Normalized FRAP (in arbitrary units) profile showing complete fluorescence recovery of the phase separated condensates at 0 h and 48 h (*n* = *3*, independent experiments). **e** Fluorescence microscopy images of (Arg)_10_ and (Asp)_10_ condensates upon mixing [2 mM (Arg)_10_ + 8 mM (Asp)_10_] in the absence of salt). Individual polypeptides (in the absence of salt) were used as controls. The scale bar is 5 μm. The experiment was repeated three times with similar observations. **f** TEM images of the condensates formed by (Arg)_10_ and (Asp)_10_ immediately after LLPS (at 0 h). *n* = *2*, independent experiments were performed. **g** ***Left panel*:** Static light scattering measurements of polypeptides at 350 nm showing the effect of increasing concentration of various additives at LLPS condition. To carry out the experiment, the LLPS mixture of (Arg)_10_ and (Asp)_10_ at *C*_*sat*_ in the presence of 10% (w/v) PEG-8000 are treated with increasing concentration of NaCl (50–150 mM), followed by 1,6 hexanediol (2-20% (w/v) and urea (0.5–2 M), respectively. ***Right panel***. DIC images showing the absence or presence of condensates in the presence of additives during the light scattering experiment. The scale bar is 5 µm. The experiment was performed two independent times with similar observations. Source data are provided as a Source Data file.

We hypothesized that if intermolecular interactions are the only necessary prerequisites for phase separation assisted by crowding, then even small polypeptides at optimum concentration can undergo LLPS, however, with different modes of interactions (hydrophobic/electrostatic/H-bonding) dictated by their amino acid sequence (Figs. 7a, 8a). To examine this, we designed a minimalistic model of 10-residue polypeptides [(Gly)_10_, (Val)_10_, (Arg)_10_, and (Asp)_10_] and characterized them using MALDI and LC-MS (Supplementary Fig. 20). We speculated that the polypeptide, (Gly)_10_, would require a very high concentration for LLPS due to lack of polyvalency/side chains by the simplest amino acid, glycine^26^. Moreover, this peptide would undergo LLPS only via intermolecular H-bonding. In contrast, (Val)_10_ might undergo intermolecular interaction based on hydrophobic interactions, which will facilitate its LLPS. Interestingly, both the peptides showed LLPS at high concentrations in 20 mM sodium phosphate buffer, pH 7.4 in the presence of 10% PEG-8000 exhibiting a varied phase regime (Fig. 7b, c). In the presence of 10% PEG-8000, (Gly)_10_ showed LLPS when peptide concentration reached ≥ 2 mM concentration, while (Val)_10_ showed LLPS ≥ 1 mM concentration (Fig. 7b–d). The (Gly)_10_ and (Val)_10_ condensates were further characterized by using fluorescence microscopy using labeled peptides (10% N-terminal NHS-Rhodamine labeled peptide + 90% unlabeled peptide). We observed condensate fusion upon contact and complete fluorescence recovery after photobleaching both at 0 h and 48 h, confirming their liquid-like property (Fig. 7e and Supplementary Fig. 21a, b). Further, the morphology of the condensates, examined using TEM revealed homogeneous electron density of the condensate state of these polypeptides (Fig. 7f). The data, therefore, suggest that small homo-polypeptide also undergo LLPS but with relatively high *C*_*sat*_ compared to other proteins under study. This indicates that intermolecular interactions between these polypeptides are much less prevalent compared to large proteins. We investigated the mode of intermolecular interaction responsible for (Gly)_10_ and (Val)_10_ LLPS using pre-formed condensate dissolution assay similar to proteins (Fig. 7g). The light scattering and DIC microscopy data suggest that the phase separation of (Gly)_10_ is majorly disrupted by the addition of 2 M urea but not by 1,6 hexanediol or NaCl (Fig. 7g). When similar experiments were performed with (Val)_10_, the condensates were disrupted only in presence of 1,6 hexanediol not in presence of either urea or NaCl (Fig. 7g). Similar observation was also obtained when we allowed both the peptides for condensate formation in presence and absence of different additives (Fig. 7h, i and Supplementary Fig. 21c). To further examine the contribution of multivalency in *C*_*sat*_, we chose glycine polypeptides. When LLPS study was performed with increasing length of polypeptide in 20 mM sodium phosphate buffer, pH 7.4 in the presence of 10% PEG-8000, we observed that (Gly)_5_, (Gly)_6_, (Gly)_7_, (Gly)_8_ and (Gly)_9_ required 40 mM, 25 mM, 20 mM, 12 mM and 8 mM concentration, respectively for their LLPS (Supplementary Fig. 21d, e). Overall, the polymer length and *C*_*sat*_ of glycine polypeptides showed a negative linear correlation (R^2^ value: 0.955), suggesting that a decrease in polypeptide length will increase the *C*_*sat*_ and vice versa (Fig. 7j). Important to note that the *C*_*sat*_ is expected to follow an exponential decay with length (or valence), but not a linear decay as per theory^63^. However, within the experimental scope, the range of glycine peptide length that was used for the study, we found a linear correlation. It might be possible that one might find an exponential decay with *C*_*sat*_ on further increasing the polymer length.

In contrast to neutral polypeptides [(Gly)_10_ and (Val)_10_], the liquid condensate formation of charged homo-polymers might occur upon neutralization of charged residues^25,34^. To examine this, we designed 10-residue polypeptides of (Arg)_10_, and (Asp)_10_ (Fig. 8a) and monitored their LLPS behavior in 20 mM sodium phosphate buffer, pH 7.4 in the presence of 10% PEG-8000. Interestingly, both the peptides showed condensate formation only in the presence of high salt as well as at high peptide concentrations (Fig. 8b, c). Our data showed that (Arg)_10_ and (Asp)_10_ formed condensates in the presence of 4 M NaCl [with 10% (w/v) PEG-8000] at a concentration of ≥ 2 mM and ≥ 8 mM, respectively (Fig. 8c and Supplementary Fig. 22a). This data suggests that at charged neutralized state, poly-Arg might possess higher polyvalency for LLPS in comparison to poly-Asp. The liquid nature of the condensates was also further characterized by fusion and FRAP studies. (Arg)_10_ condensates showed fusion upon contact to form larger condensates and all the polypeptide condensates showed complete fluorescence recovery after photobleaching, confirming their liquid-like property at both 0 h and 48 h (Fig. 8d and Supplementary Fig. 22b, c). To further examine whether intermolecular interactions between oppositely charged polypeptides facilitate LLPS, we monitored the co-LLPS of (Arg)_10_ and (Asp)_10_. When two oppositely charged polypeptides were mixed at their *C*_*sat*_ (Fig. 8e and Supplementary Fig. 22d) as well as with different ratios (Supplementary Fig. 22f), we observed spontaneous phase separation in the absence of salt. We found the *C*_*sat*_ for co-LLPS reached 0.75 mM for both peptides when mixed together, suggesting that charge neutralization favors their co-LLPS (Supplementary Fig. 22f). In identical conditions, however, the individual polypeptides did not show any LLPS (Fig. 8e and Supplementary Fig. 22d). Further, the morphology of the condensates was examined using TEM, which revealed homogeneous electron density of the condensate state of these polypeptides (Fig. 8f). We further investigated the nature of interaction responsible for LLPS of charged polypeptides using preformed condensate using light scattering and DIC imaging (Fig. 8g). We observed that LLPS of (Arg)_10_ was majorly disrupted by the addition of 20% (w/v) 1,6 hexanediol; whereas dissolution of (Asp)_10_ condensates were observed on addition of 2 M urea. This suggests that upon charge neutralization, (Arg)_10_ and (Asp)_10_ polypeptides undergo LLPS through hydrophobic and H-bonding interaction. A similar observation was also obtained when additives were added before phase separation and the condensate formation was examined using DIC microscopy (Supplementary Fig. 22e).

## Discussion

Increasing evidence underscores the ability of condensate formation by a wide range of proteins either related to membrane-less organelles formation for performing a normal cellular function^1,7–9^ or as a nucleation center for protein aggregation^17–22^. LLPS might be tightly regulated based on the protein localization in specific organelles where it performs its native function^1,5,22,78–80^ and the presence of DNA/RNA or other co-factors in cell^24,38,78,81–86^. Recent studies indicated that the condensate state might be a proteome-wide phenomenon^33^ and may be considered as the fundamental state of proteins^87^, besides the native and the amyloid state^88,89^. Also, several recent studies have provided prediction tools and physical frameworks in encoding the molecular grammar driving condensate formation by a wide range of proteins^23,27,30,90^. In a given experimental condition, although proteins (both folded and disordered) and polypeptides form liquid condensates, the underlying driving forces resulting in the formation of condensate remain unclear. Phase separation is thermodynamically favored when a protein has enough concentration and adequate interaction strength^87^. The thermodynamics of condensate formation is a complex interplay between entropy and enthalpy, where the decrease in entropy (ΔS) due to molecular clustering must be overcome by the increase in enthalpy (ΔH), which generally is achieved through intermolecular interactions^64,65^. The weak multivalent interactions hold the higher-order molecular arrangement inside the condensate and maintain the liquid-like property. These include cation-π, π-π interaction, charge dipole and hydrophobic interactions^32,91^.

It has been well-documented that low-complexity, intrinsically disordered, or prion-like domains promote LLPS^23–28^. Due to the lack of a specific fold, these domains generally provide more multivalency for intermolecular clustering, a prerequisite for LLPS. Further, the intrinsically disordered proteins are also known to bind to different partners for functionality through a short segment while a significant stretch of the protein maintains the overall conformational flexibility of the complex^92^. The binding interaction of such fuzzy proteins may also result in protein assemblies responsible for biomolecular condensates^90,92,93^. On the other hand, multidomain globular proteins can also facilitate intermolecular contacts due to multiple-binding sites (similar to patchy colloids^94,95^) and can form liquid condensates in a suitable reaction condition. However, the chances and strength of both specific and non-specific intermolecular interactions can be further enhanced with an increase in protein concentration (or due to crowding) which may favor LLPS. In this concentration regime, the inter-molecular ordering or self-assembly state is more thermodynamically favorable over individual protein molecules. At low concentrations, proteins, however, are more diffusive in nature with no intermolecular ordering. Thus, the concentration of a protein molecule finely balances the intermolecular interaction and determines the collective behavior of proteins^87^. Indeed, we observed that various proteins with diverse sequences and structures could readily undergo LLPS, however, with a wide variation in their *C*_*sat*_ and kinetics (Fig. 1). This is expected as the nature of amino acids and their pattering^96–98^ in three-dimensional space would dictate the extent of intermolecular interaction determining the *C*_*sat*_. The growth kinetic study further indicates that all proteins above the *C*_*sat*_ give rise to a liquid condensate state after a lag time. Understanding the nucleation mechanism of LLPS, which is otherwise a thermodynamically uphill process, is crucial and an ongoing research area. Recently, Martin et al. have found a multistep nucleation process prior to detectable LLPS where small complexes in the nanoscale size distribution are formed even in the sub-critical protein concentration^99^. After the initial complex formation, which is an energetically unfavorable process, the monomer starts to recruit in the complex with high affinity to form the mesoscale clusters^100^, which can further grow by classical homogeneous nucleation. Recent studies also further suggested that at sub-saturation concentrations, some proteins form nanoclusters^50,101^. The presence of nanoclusters at sub-saturation concentration and its increase in size with concentration suggest that many proteins might phase separate through nanocluster formation, which could be even present after macroscopic phase separation as shown for α-Syn^50^. Although, currently the relationship between nanocluster formation below the saturation concentration and macroscopic phase separation above saturation concentration is not known as there is a possibility that the nanocluster formation also could be linked with macroscopic condensate formation for a particular protein (could be encoded by protein sequence). Apart from the nanoclusters, another “mesoscopic” protein condensate of several hundred nanometers in size and liquid-like clusters has been observed for many proteins in different experimental conditions^102–105^. The mechanism of transient complex formation prior to mesoscopic condensate formation has been explained as a common feature for several biomolecules^104^. We speculate that although LLPS of proteins of various structures and sequences are detectable above saturation concentration, the initiation of the cluster formation might start even below the saturation concentration and the subsequent growth of the clusters might be modulated by the intermolecular interaction of the proteins, which results in different saturation concentration for the detectable LLPS. However, this needs further investigation. Important to note that the condensates formed in our case are above the saturation concentration, as the size of the condensates is much higher than the size of the nanocluster observed below the saturation concentration^50^. Although the nanocluster formation and the presence of small condensates, which might not be sedimented by ultracentrifugation or not visible under the confocal microscope (limit is 500 nm) might also affect the saturation concentration determination.

A major theory that is often used to explain phase separation is the FH theory^63^. Although FH theory does not account for nanocluster formation, it takes into account the Flory parameter χ, which represents the strength of the monomer-solvent interaction averaged over the protein^64,65^. Hence, the implication of possible nanoclusters formation by protein and its effect on *C*_*sat*_ is not clearly apparent at present and is beyond the scope of FH theory.

We have shown that both folded and disordered proteins can form reversible, dynamic condensates in a concentration-dependent manner. Interestingly, the phase separation by proteins does not require a misfolding or drastic structural transition, suggesting that a high enough concentration (or factors promoting intermolecular interaction) is sufficient for inducing LLPS (Figs. 3 and 5). Consistent with all previous studies^17,18,20^, most LLPS systems maintain their liquid-like nature; upon aging, however, a subset of proteins indeed shows a certain extent of viscoelastic transition (partial rigidification) (Fig. 4). We found that gradual rigidification does not mandatorily corroborate with amyloid fibril formation. The viscoelastic transition of liquid condensate might also occur due to crystal-like packing/ amorphous aggregation in the dense LLPS milieu^1,2,6,22^. This suggests that rigidification of liquid condensates might be specific to proteins with respect to sequence/structure and could preserve the structure (therefore protein function) of most of the proteins^1,2,6,22,60^. In this context, mesoscopic clusters by p53 mutant protein were also reported to promote the essential sites of nucleation for higher-ordered solid condensates such as misfolded protein aggregates forming amyloid fibrils apart from macroscopic protein-rich condensates^105^.

There might be a possibility that the cellular environment, sequence and structure of protein might dictate protein aggregation either from liquid condensate and/or small/large mesoscopic clusters. Moreover, there can also be a rearrangement of existing molecular machineries and component systems in cells that may give rise to condensates of a few tens of nanometers in diameter^37,106^.

Although most of the proteins under study harness a combination of intermolecular interactions for their condensate formation and stability (Fig. 5), it is apparent that the driving forces are complex and diverse, and multiple factors can determine *C*_*sat*_. Moreover, hydrophobic interaction also showed an important role in many proteins/peptides phase separation under study, consistent with previous studies of proteins LLPS^18,70,107,108^. It seems that H-bonding interaction also promotes phase separation for globular proteins where electrostatic and/or hydrophobic interaction sites are less prevalent. Interestingly, our designed peptide condensate data clearly showed that (Gly)_10_ and (Asp)_10_ (in the presence of NaCl), undergo condensate formation using H-bonding interaction, which requires much more *C*_*sat*_ than (Val)_10_ and/or (Arg)_10_ where we observed hydrophobic interaction playing a major role. Therefore, proteins/peptides undergoing LLPS through H-bonding require much more concentration so that enough interactions are made possible for network formation in the confined space for condensate formation.

Further, the *C*_*sat*_ for LLPS also shown to be strongly correlated with their intermolecular protein-protein interaction strength (Fig. 6c). It is also dictated by the molecular weight (polymer length/amino acid number in proteins) and the nature of amino acid side chains^6,11,29,36,91^. For example, a stretch of a glycine-rich polypeptide with higher polypeptide flexibility and the absence of sidechain polyvalency might decrease the extent of intermolecular interaction^26^. However, hydrophobic amino acid (Val) and other aromatic amino acids might increase the interaction strength due to hydrophobic and other interactions (such as cation-π)^23,26,31^ when present in proteins. This interaction strength is highly reflected in *C*_*sat*_ as (Gly)_10_ requires double the polypeptide concentration (2 mM) for LLPS in comparison to (Val)_10_ (1 mM). Further, homopolymers of charged amino acids might not undergo LLPS due to charge-repulsion unless their charges are neutralized^34^. Indeed, our data showed that (Arg)_10_ and (Asp)_10_ homopolymers undergo LLPS either in the presence of salt^24,38,40,81,84^ (Fig. 8b) or when they are mixed (Fig. 8e).

Important to note that the in vivo LLPS depends on other factors, for example, the presence of other biomolecules or microenvironment. It is possible that the active cellular processes might modify the crowded milieu, and hence, maintain the protein solubility^36^. Therefore, the present study and its relevance to in vivo at this point is not clear and need further investigation. Since, in vivo LLPS is most likely a multi-component system, which might not be applicable to our single-component experimental conditions. However, the present study indicates that in a given condition, protein/peptide in general might phase separate, irrespective of the relevance in in vivo system as the condition of in vivo and in vitro might differ significantly.

In conclusion, our study suggests that proteins/polypeptides with different structures and sequences can undergo LLPS although with different apparent *C*_*sat*_ (Supplementary Fig. 23). The presence of IDRs might provide an advantage in undergoing phase separation as they have higher polyvalency as well as a low structural order, resulting in substantially a greater number of molecular interactions^1,6,14,18,23^. However, this phenomenon can be protein specific (with specific sequence and structure) but might not be applicable to all proteins/peptides in general (Supplementary Fig. 2). Moreover, once a protein undergoes LLPS, its subsequent rigidification might require very high concentration and/or specific interactions. Deregulation of protein quality control and turnover mechanisms in cells might pave the way for aberrant phase transition^1,6,12,109^. A similar generic state hypothesis has also been proposed for amyloid fibril^110^ formation by proteins and polypeptides with an argument that cellular/subcellular conditions, protein quality control machinery and protein expression/post-translational modification do not allow such transition in cells. Also, nature perhaps has evolved with a ‘negative design’ for proteins, which prevents amyloidogenesis^111^.

## Methods

All the reagents and chemicals used for the study were purchased from Sigma (USA) unless mentioned otherwise. The product information of the proteins is provided in Supplementary Tables 1 and 2. NHS-Rhodamine (Catalog no. 46406), and Fluorescein-5 isothiocyanate (FITC) (Catalog no. F1906) were procured from ThermoFisher Scientific (USA). FITC-PEG-COOH, molecular weight 5000 (Catalog no. PHB-3925) was purchased from Creative PEG Works (North Carolina, US). The protease inhibitor cocktail (PIC) was obtained from Roche Applied Science (Catalog no. 05056489001). 1-Hydroxybenzotriazole hydrate (HOBt) (Catalog no. 157260), Triisopropylsilane (TIPS) (Catalog no. 233781), Trifluoroacetic acid (TFA) (Catalog no. T6508), *N, N*’-Diisopropylcarbodiimide (DIC) (Catalog no. D4781), and Polyethylene glycol molecular weight 300 (Catalog no. 202371-5 G) were purchased from Sigma (USA). *N, N*-Dimethylformamide (DMF) (Catalog no. 8.22275.2521), Dichloromethane (DCM) (Catalog no. 1.94508.2521), Acetonitrile (ACN) (Catalog no. 60003025001730), and Diethyl ether (Catalog no. 1.07026.0521) were purchased from Merck Millipore. Wang resin (100–200 mesh, 0.7 mmol/ g) (Catalog no. 8.55002), and 4-(Dimethylamino) pyridine (DMAP) (Catalog no. 8.51055) were purchased from Novabiochem (Germany). The polypeptides, pentaglycine (Catalog no. G5755), and hexaglycine (Catalog no. G5630) were purchased from Sigma-Aldrich (USA).

### In silico analysis of proteins

The FASTA sequence of all proteins was obtained from Uniprot (Supplementary Table 1). These protein sequences were used for various in silico analyses. The online tool IUPred2A^45^ was used for the identification of the disordered regions for all proteins using the amino acid sequence as input. It provides a score between 0 and 1 for each amino acid residue, which corresponds to the probability of the residue being part of a disordered region. SMART^46^ (Simple Modular Architecture Research Tool) identifies and annotates the presence of low-complexity regions from the amino acid sequence. The LLPS propensity was predicted using the catGRANULE^47^ algorithm. The propensity score was determined and plotted for all the proteins. PONDR^48^ is an algorithm used for predicting the naturally disordered region. The percentage of disorderness for all proteins was determined using PONDR (VLXT predictor), which was plotted against *C*_*sat*_. All the data was plotted using OriginPro 2021 (Origin Lab, USA) software.

### Expression and purification of α-synuclein (α-Syn) and Tau protein

α-Syn was expressed and purified using previously established protocols with slight modifications^112,113^. Briefly, competent *E. coli* BL21 (DE3) cells were transformed using cloned plasmid and the expression was induced using isopropyl-β-D-thiogalactoside (IPTG) (1 mM). Following this, the cells were centrifuged at 1699 x g for 30 min at 4 °C. The pellet was resuspended in lysis buffer (50 mM Tris, 10 mM EDTA, 150 mM NaCl) and PIC (Roche) was added to prevent proteolytic cleavage. The cells were further lysed using a probe sonicator (Sonics & Materials Inc.) at 40% amplitude with 3 s ON and 1 s OFF pulse for 10 min. The solution was then heated at 95 °C for 20 min and centrifuged at 8603 x g for 30 min. The supernatant was used for nucleic acid precipitation using 10% streptomycin sulfate (136 μl/ml) and glacial acetic acid (228 μl/ml). The solution was then centrifuged at 8603 x g for 30 min at 4 °C to remove nucleic acid. Following this, the protein precipitation was carried out using saturated ammonium sulfate (equal volume). The solution was kept at 4 °C for 4 h for complete precipitation and centrifuged at 10621 x g for 30 min at 4 °C. The protein was further washed using ammonium sulfate solution (50%) and centrifuged at 10621 x g. Finally, the protein was washed using ammonium acetate (100 mM) and precipitated using ethanol. This step was repeated three times. The solution was centrifuged and the pellet was dissolved in a minimum volume of ammonium acetate (100 mM) and lyophilized. The lyophilized protein was redissolved in 20 mM sodium phosphate buffer, pH 7.4, and further purified using size exclusion chromatography (SEC) in the Q Sepharose column before the LLPS experiment. The purity of the protein was confirmed by SDS-PAGE and Coomassie blue staining method.

Expression of full-length wild-type Tau protein (2N4R isoform containing 441 residues) was carried out by transforming tau/pET29b plasmid (Addgene id 16316) into *E. coli* BL21 (DE3) competent cells. The expression and purification protocol of Tau protein were similar to α-Syn with minor modifications. Briefly, bacterial cells were grown in the presence of Kanamycin in Luria broth (LB) media at 37 °C to an optical density value between 0.7-1. Protein expression was induced with 1 mM IPTG followed by 4 h incubation at 37 °C in 200 rpm rotation. Cells were harvested by centrifugation and resuspended in 60 ml of lysis buffer (50 mM Tris, 10 mM EDTA, and 150 mM NaCl, 5 mM DTT at pH 8.0). PIC was added to the lysis buffer to prevent proteolytic cleavage. The cells were lysed by sonication (40% amplitude, 3 s ON and 1 s OFF) for 15 min using a probe sonicator (Sonics and Materials Inc., USA) and heat-denatured in hot water at 95 °C for 20 min. Cell debris and other denatured proteins were pelleted down by centrifugation at 10621 x g, 4 °C for 30 min. DNA was precipitated from the supernatant using streptomycin sulfate [10% (w/v)] and glacial acetic acid. After DNA removal, an equal volume of saturated ammonium sulfate was added and incubated at 4 °C overnight for protein precipitation. The solution was centrifuged twice at 15294 x g, 4 °C for 30 min. Pellet was dissolved in 100 mM ammonium acetate and reprecipitated in an equal volume of ethanol. The final pellet was redissolved in a minimum volume of 100 mM ammonium acetate, flash-frozen with liquid nitrogen, and lyophilized. The lyophilized protein powder was stored at -20 °C until used for experiments. The required amount of protein was dissolved in equilibrating buffer (20 mM sodium phosphate buffer, 1 mM DTT) and further purified by size exclusion chromatography in the Q Sepharose column before the experiment. The purity of the protein was confirmed by the standard SDS-PAGE and Coomassie blue staining method.

### Size exclusion chromatography (SEC) of proteins

All the commercially purchased and recombinantly expressed/purified (α-Syn and Tau) proteins were dissolved in a filtered 20 mM sodium phosphate buffer (pH 7.4, 0.01% sodium azide). The Superdex 200 TM 10/300 SEC column was pre-equilibrated with 3 column volumes of 20 mM sodium phosphate buffer (pH 7.4, 0.01% sodium azide) and the protein solutions were injected into the column. The proteins were isolated and the purity of the protein from SEC was confirmed using SDS-PAGE. The protein concentrations were determined by Beer-Lamberts law (c = *A*/*εl*), where *c* is the protein concentration in molar, *l* is the path length in cm, *A* is the absorbance value at the respective wavelength, and *ε* is the molar absorption coefficient at the respective wavelength, using UV spectroscopy (Jasco V650, Japan). The absorbance measurement at 280 (A_280_) was used for determining the protein concentration for all the proteins except the chromophore-containing proteins such as Hb (A_406_, ε_406_ = 270548 M^−1^cm^−1^)^114^, Mb (A_408_, ε_408_ = 129000 M^−1^cm^−1^)^115^, Cyt c (A_410_, ε_410_ = 101600 M^−1^cm^−1^)^116^, and CATA (A_405_, ε_405_ = 324000 M^−1^cm^−1^)^117^ whose protein concentration was determined using the extinction coefficient of the respective chromophore group.

### Solid-phase peptide synthesis

All the peptides were synthesized by 9-fluorenylmethoxy- carbonyl (FMOC) chemistry using the manual solid-phase peptide synthesis method^118^. The synthesis was performed with a scale of 0.20–0.25 mmol on a Wang resin. In a typical synthesis, the first amino acid was loaded on Wang resin by dissolving 1 eq. of amino acid and HOBt in DMF, followed by the addition of 1 eq. of DIC and finally DMAP in catalytic amt. (0.1 eq.). The coupling was kept for 2-3 h and washed several times with DMF and DCM after the completion of the reaction. The FMOC group was removed using 25% piperidine in DMF. The next coupling was repeated using DIC/HOBt coupling agent with the equivalent amount of the next amino acid. After the synthesis of the desired length polypeptide, the peptide was cleaved off from the resin using a standard cleavage cocktail, TFA: Phenol: TIPS: water (88:5:2:5). Further, the cleavage solution was transferred into an ice-cold ether solution to get the precipitated peptides. After precipitation, the ether solution was evaporated and the peptides were redissolved in ammonium bicarbonate (50 mM).

The synthesized peptides [(Gly)_10_, (Asp)_10_, and (Arg)_10_] were purified using HPLC equipped with a reverse phase-C18 column. The mobile phase was used with the 90 min gradient system starting from 10% ACN/water (0.1% TFA) to 90% ACN/water system with a flow rate of 1 ml/min. The samples were injected from a 5 mg/ml stock concentration and 200 μl of peptide aliquot solution was injected using an autosampler injector. The instrument was provided with a UV-Vis detector (dual-wavelength) and absorbance at 195 nm was recorded. For analysis, we used the data acquired at 195 nm (the analytes had maximum molar absorptivity). Using these parameters, all the synthesized polypeptides were separated. However, (Val)_10_ was not purified using HPLC, since it exhibits poor solubility in the given HPLC mobile phase gradient system and therefore, was used as synthesized. The purified polypeptides were characterized using ESI LC-MS and MALDI analysis.

### Fluorescent labeling of protein/peptide

The NHS-Rhodamine and FITC labeling of protein/peptides was done as per the manufacturer’s protocol (ThermoFisher Scientific, USA). Briefly, 5X molar excess of FITC/rhodamine (dissolved in DMSO) was added to the protein obtained after SEC. For FITC, the mixture was incubated on a magnetic stirrer at 4 °C for 6 h in the dark with slow rotation. For NHS-Rhodamine labeling, the protein mixture was incubated for 2 h at room temperature in the dark with slow stirring. The excess dye was removed by dialysis using different molecular weight cut-off membranes depending upon the molecular weight of the proteins in 20 mM sodium phosphate buffer (pH 7.4) at 4 °C for 48 h, with regular buffer exchange in 6 h intervals. The concentration of the labeled protein was determined as per the manufacturer’s protocol. The polypeptides were labeled as mentioned previously. The excess FITC/rhodamine dye was removed by dialysis in 20 mM sodium phosphate buffer (pH 7.4) for 12 h with regular buffer exchange with 2 h intervals at 4 °C. After dialysis, the labeled polypeptide solution was lyophilized and the concentration was determined by redissolving the dry weight in 20 mM sodium phosphate buffer (pH 7.4). For all experiments, we used 1:10 (v/v) of labeled versus unlabeled protein/polypeptide, unless mentioned otherwise.

### In vitro liquid-liquid phase separation of proteins and peptides

For LLPS experiments, acid-treated coverslips were used^40^. To do this, the glass slides and 12 mm coverslips (Blue Star, India) were kept in aqua regia [1:3 (v/v) nitric acid/hydrochloric acid] for 12 h and thoroughly washed with Milli-Q. After every wash, the pH of the Milli-Q was checked until it reached 7.0. The slides and coverslips were air-dried in a laminar air-flow hood under sterile conditions and used for all the subsequent LLPS experiments.

For LLPS, the proteins purified using SEC were used to prepare the reaction mixture at different protein and PEG-8000 concentrations [(0%, 5% 10%, 15% and 20% (w/v)] in 20 mM sodium phosphate buffer (pH 7.4, 0.01% sodium azide) to determine the phase regime. Similarly, LLPS experiments were also performed in the presence of PEG-300 to study the effect of PEG length in phase separation. For this, the purified proteins after size exclusion chromatography at their respective *C*_*sat*_ were used to prepare the reaction mixture in the presence of 10% (v/v) PEG-300 in 20 mM sodium phosphate buffer (pH 7.4). Moreover, proteins were tested for LLPS in the absence of PEG-8000 under different conditions (high concentration, addition of NaCl and/or change in pH) in 20 mM sodium phosphate buffer, pH 7.4. The experiment was repeated two times.

The polypeptides (Arg)_10_ and (Asp)_10_ were dissolved in 20 mM sodium phosphate buffer (pH 7.4). For (Val)_10_ and (Gly)_10_, 1 mg of the respective polypeptide was dissolved in 20 μl of TFA to obtain a homogenous solution and the volume was adjusted to 50 µl by the addition of 20 mM sodium phosphate buffer (pH 7.4). TFA was removed by nitrogen gas purging and 20 mM sodium phosphate buffer was added to obtain a stock solution of 5 mM for both polypeptides. For LLPS of polypeptides, the reaction mixture at different polypeptide concentrations and PEG-8000 concentrations [(0%, 5% 10%, 15% and 20% (w/v)] in 20 mM sodium phosphate buffer (pH 7.4, 0.01% sodium azide) was prepared to determine the phase regime. The reaction mixture (proteins/polypeptides) was drop-casted on the acid-treated slides and sandwiched with an acid-treated 12 mm glass coverslip (Blue Star, India). The coverslips were sealed using commercially available nail paint. The slides were incubated at 37 °C in a moist chamber and phase separation was monitored using 63X oil immersion objective in the DIC (Differential Interference contrast) mode and fluorescence mode under a DMi8 microscope (Leica Microsystems, Germany). All the images were obtained at 16-bit depth with 2048 × 2048 pixels resolution unless mentioned otherwise. The images were analyzed using ImageJ (NIH, Bethesda, USA) software.

For co-LLPS of peptides, FITC labeled (Asp)_10_ was mixed with NHS-Rhodamine labeled (Arg)_10_ in the presence of PEG-8000 (10% w/v) in 20 mM sodium phosphate buffer (pH 7.4, 0.01% sodium azide) at various peptide concentration ratios. For all the experiments, we used 1:10 (v/v) of labeled versus unlabeled peptides, unless mentioned otherwise. The mixture was drop-casted on acid-treated glass slides and sandwiched with a 12 mm acid-treated coverslip. The coverslip was sealed using commercially available nail paint. These were used during the microscope image acquisition of co-LLPS condensates. The images were processed using ImageJ (NIH, Bethesda, USA) software.

### Determination of PEG partitioning into condensates

The partitioning of PEG inside condensates was monitored using FITC-labeled PEG-5000. For LLPS, a subset of NHS-Rhodamine labeled proteins at their respective *C*_*sat*_ were mixed in the presence of 10% PEG (5% (w/v) FITC-labeled PEG-5000 + 5% (w/v) PEG-8000) in 20 mM phosphate buffer (pH 7.4). Similarly, in another case, a subset of proteins at *C*_*sat*_ was allowed to undergo LLPS in the presence of PEG-8000, in which immediately after LLPS, 5% (w/v) FITC labeled PEG-5000 was added to verify PEG partitioning. The condensate formation was observed using a confocal microscope (LSM 780 Zeiss Axio-Observer Z1 microscope (inverted)) equipped with iPlan-apochromat 63X/1.4 NA oil immersion objective and with an appropriate fluorescence channel. The images were obtained with a frame size of 1024 pixels x 1024 pixels with 8 bit-depth unless mentioned otherwise. The images were processed using ImageJ (NIH, Bethesda, USA) software. The experiment was repeated two times.

Thereafter, the apparent partition coefficient of PEG into the condensates was calculated using, $\frac{{PE}{G}_{{inside}}}{{PE}{G}_{{outside}}}$. For example, in the case of β-lac the average fluorescence intensity of ${PE}{G}_{{inside}}$ was 7.63 (A.U.) and ${PE}{G}_{{outside}}$ was 404.34 (A.U.). The number of condensates used for fluorescence intensity determination was >50. Therefore, the apparent partition coefficient is $\frac{{PE}{G}_{{inside}}}{{PE}{G}_{{outside}}}$ = 7.63 (A.U.)/404.34 (A.U.) = 0.01 = ~0 for β-lac. Similar calculations were done for the other seven proteins and the apparent partition coefficient was ~0.

### Fluorescence and confocal microscopy

The in vitro liquid condensate formation for all the NHS-Rhodamine labeled proteins and peptides [1:10 (v/v) labeled to unlabeled protein/peptide] were observed using a DMi8 microscope (Leica Microsystems, Germany) under DIC and fluorescence mode using an appropriate fluorescence channel (560 nm/488 nm) at 16-bit depth with 2048 × 2048 pixels resolution. The FRAP analysis at *C*_*sat*_ of proteins (0 h and 48 h after LLPS) were performed using a laser scanning confocal microscope (LSM 780 Zeiss Axio-Observer Z1 microscope (inverted)) equipped with iPlan-apochromat 63X/1.4 NA oil immersion objective and with appropriate fluorescence channel (560 nm). The images were obtained with a frame size of 512 pixels X 512 pixels with 8 bit-depth unless mentioned otherwise. The images were processed using ImageJ (NIH, Bethesda, USA) software.

### Preparation of HeLa cell cytoplasmic extract

HeLa cells (authenticated cell line procured from NCCS cell repository, Pune, India) were used for the preparation of the cytoplasmic extract (CE) for the study as per previously established protocol^54,119^. The cell density of 5 × 10^5^/mL was used such that the cell confluency was less than 80%. The cells were washed three times with PBS and were harvested using trypsin. The cell solution was incubated in hypotonic buffer [20 mM Tris-HCl (pH 7.4), 10 mM KCl, 2 mM MgCl_2_, 1 mM EGTA, 0.5 mM DTT and 0.5 mM PMSF] for 3 mins at 4 °C to enhance fractionation. NP-40 (0.1%) was added to the cells and incubated for 3 mins for membrane lysis. The cell suspension was then centrifuged at ~1000 x g for 5 mins at 4 °C. The supernatant containing the cytoplasmic extract was collected and centrifuged at ~15,000 x g for 3 mins at 4 °C for removing the debris. The supernatant was collected and dialyzed with 20 mM sodium phosphate buffer (pH 7.4) at 4 °C for 6 h using a 1 kDa cut-off membrane. The total protein concentration was determined using Bradford’s protein estimation assay. For the LLPS study, all the proteins were mixed with CE (10 mg/mL protein concentration) at their respective *C*_*sat*_ using NHS-Rhodamine labeled [10% (v/v) labeled to unlabeled] proteins. The condensate formation was observed using a DMi8 microscope (Leica Microsystems, Germany) under DIC and fluorescence mode using an appropriate fluorescence channel (560 nm) at 16-bit depth with 2048 × 2048 pixels resolution.

### Determination of *C*_*sat*_ by centrifugation

The SEC purified protein samples at approximately three to four times the respective *C*_*sat*_ (determined through microscopic observation) in the presence of PEG-8000 (10% w/v) were incubated in an eppendorf tube at 37 °C in a moist chamber for LLPS. Immediately after LLPS, the samples were centrifuged at ~1,50,000 x g for 30 mins using ultracentrifugation (Beckman Coulter Optima^TM^ Max-XP, USA). 20 μL of the supernatant was taken, diluted in 20 mM sodium phosphate buffer (pH 7.4) and absorbance at 280 (A_280_) was measured using UV spectroscopy (Jasco V650, Japan). The absorbance at 280 nm was used for determining the concentration of the dilute phase of all the proteins except the chromophore-containing proteins such as Hb (A_406_, ε_406_ = 270548 M^−1^cm^−1^), Mb (A_408_, ε_408_ = 129000 M^−1^cm^−1^), Cyt c (A_410_, ε_410_ = 101600 M^−1^cm^−1^), and CATA (A_405_, ε_405_ = 324000 M^−1^cm^−1^) whose protein concentration was determined using the extinction coefficient of the respective chromophore group. The concentration in the dilute phase after phase separation was considered as *C*_*sat*_,^120^ which is consistent with the microscopic observation.

### Light scattering measurements

The saturation concentration (*C*_*sat*_) for LLPS of the respective protein sample in the presence of 10% PEG-8000 (w/v) (LLPS-inducing condition) was used for static light scattering (SLS) measurements. The excitation and emission wavelength were set at 350 nm and the slit width was kept at 5 nm for both. The measurements were acquired in continuous mode using a spectrofluorometer (JASCO FP 8500, USA). The experiment was performed twice. A plot of light scattering intensity against time was plotted, which resulted in a sigmoidal curve. The data were background corrected, normalized, and fitted using the Boltzmann equation and t_1/2_ was calculated as follows;

3 $$y={y}_{0}+({y}_{\max }-{y}_{0})/[1+{e}^{-\left(k\right.(t-{t}_{1/2})}]$$

Where, *y* = the light scattering intensity at a particular time point, *y*_*max*_ = maximum light scattering intensity, *y*_*0*_ = light scattering values at *t*_*0*_. The data was plotted using OriginPro 2021 (Origin Lab, USA) software. The t_1/2_ was determined using the Eq. 3 and the graph was plotted using the GraphPad Prism 8 software.

To investigate the nature of intermolecular interactions responsible for protein LLPS, a sequential titration assay was performed using static light scattering at 350 nm. To do so, NaCl (disrupts electrostatic interaction), 1,6 hexanediol (disrupts hydrophobic interaction) and urea (disrupts H-bonding and van der Waals forces) stock solutions were prepared. 100 μl of SEC purified protein samples at their respective *C*_*sat*_ in the presence of 10% (w/v) PEG-8000 were incubated in an Eppendorf at 37 °C in a moist chamber for LLPS. Immediately after LLPS (0 h), the samples were sequentially titrated with increasing concentration of NaCl (50-150 mM) followed by 1,6 hexanediol (2-15% w/v) and urea (0.5-2 M). Important to note that NaCl was added initially to the pre-formed condensates followed by 1,6 hexanediol and urea. The 1,6 hexanediol experiments had NaCl in them and the urea experiments already had both NaCl and 1,6 hexanediol in them. Considering the volume of work, the dissolution assay using a scrambled sequence of the additives was performed only for LT, BSA and β-cas. After the addition of each concentration of the additives, the light scattering was recorded using a spectrofluorometer for 30 s and the value at 15th s was used for data analysis. For studying the kinetics of LLPS in the presence of additives, the protein samples at their respective *C*_*sat*_ in the presence of 10% (w/v) PEG-8000 and NaCl (150 mM) or 1,6-hexanediol (10%) (w/v) was used for the measurements. The light scattering intensity against time was plotted in OriginPro 2021 (Origin Lab, USA) software. Two independent experiments were performed for this assay.

### Fluorescence Recovery After Photobleaching (FRAP)

For FRAP experiments, NHS-rhodamine labeled [10% labeled and 90% unlabeled (v/v)] protein/peptides mixture in the presence of PEG-8000 (10% w/v) or cytoplasmic extract at respective *C*_*sat*_ were incubated in Eppendorf at 37 °C in a moist chamber for LLPS. At different time points (0 h and 48 h) the samples were drop-casted on acid-treated glass slides and covered with 12 mm acid-treated coverslip. The condensate was bleached and fluorescence recovery was determined using a previously established protocol^18^. The experiments were performed using a built-in FRAP module in Zeiss Axio-Observer Z1 confocal microscope with 63X oil-immersion objective (NA 1.4). A 561 nm DPSS 561-10 laser (at 100% laser power) was used to bleach the center of the condensate and two other regions of interest (ROI) with the same diameter were also recorded to determine the background and passive bleaching corrections. The fluorescence intensity after bleaching was simultaneously recorded for all three ROIs using the Zen Pro 2011 (Zeiss, Germany) software provided with the instrument. The images were obtained with a frame size of 512 pixels x 512 pixels with 8 bit-depth. The fluorescence recovery data were background corrected, normalized, and fitted using the single exponential recovery function in OriginPro 2021 (Origin Lab, USA) software, and t_1/2_ was determined. The equation used for fitting is as follows^17,121–124^;

4 $$I\left(t\right)=A\left(1-\exp \left(\frac{-t}{\tau }\right)\right)+C$$

Where, τ is the fluorescence recovery time constant, ‘A’ corresponds to the mobile fraction of the fluorescent probe, and ‘*C*’ is the Y-intercept of the recovery curve.

The half-time of the recovery (*t*_*1/2*_) was calculated from,

5 $${t}_{\frac{1}{2}}=\tau {{{{\mathrm{ln}}}}}\left(2\right)$$

The graph was plotted using the OriginPro 2021 (Origin Lab, USA) software.

### Thioflavin T (ThT) fluorescence assay

For the ThT fluorescence assay, 100 µl of unlabeled SEC isolated protein samples and the proteins which showed low recovery of fluorescence at 48 h after LLPS; (β-cas, CATA, GG, LT, Tau, and α-Syn) were incubated at 37 °C for LLPS (0 h and 48 h). At both time points, the sample was diluted in 20 mM sodium phosphate buffer (pH 7.4, 0.01% sodium azide) to a final concentration of 10 µΜ and ThT fluorescence assay was performed. To do that 1 µl of 1 mM ThT dye (prepared in 10 mM Tris-HCl buffer, pH 8.0, 0.01% sodium azide) was added to the protein samples. ThT fluorescence measurements were recorded using Spectrofluorimeter (JASCO FP 8500, USA) instrument at an excitation wavelength of 450 nm and an emission range of 460-500 nm with a slit width of 5 nm for both excitation and emission measurement. The graph was plotted using the GraphPad Prism 8 software at the emission maxima (λ_max_ ~ 480 nm) after background corrections. The experiment was repeated two times.

### Transmission Electron Microscopy (TEM)

TEM analysis was performed for a subset of proteins (proteins that showed substantial rigidification using FRAP data; α-Syn, LT, GG, Tau, β-cas, and CATA) at their respective *C*_*sat*_ immediately after LLPS (0 h) and after 48 h of incubation. For sample preparation, the coverslip containing LLPS solution was removed from the slide of LLPS samples (0 h and 48 h) and it was directly transferred on the EM grid (Electron Microscopy Sciences, USA), incubated for 5 min. The grids were stained using uranyl formate (1% w/v) for 5 min and excess dye was removed with the help of filter paper. The grids were directly air-dried without any further washes before imaging. Imaging was done using JEOL Field Emission Gun-transmission electron microscopy (JEM 2100 F, JEOL, Japan) at 200 kV with X10000 magnification. The images were recorded digitally using the Gatan microscopy suite® (Gatan, USA).

### 8-anilino-1-naphthalenesulfonic acid (ANS) binding assay

To determine the extent of the exposed hydrophobic surface of proteins, ANS fluorescence binding assay was performed. Briefly, 3 µl of 5 mM ANS (prepared in 20 mM phosphate buffer, pH 7.4) was added to 10 µM of 100 µl of all the SEC purified protein samples in the presence of PEG-8000 (10% w/v) in 20 mM sodium phosphate buffer (pH 7.4, 0.01% sodium azide). The mixture was incubated for 5 min in the dark at room temperature. The fluorescence intensity measurements were done using a spectrofluorimeter (JASCO FP 8500, USA) with 370 nm as an excitation wavelength and 400-600 nm as an emission wavelength range. The slit width was set to 5 nm for both excitation and emission wavelength. The acquired spectral intensities were plotted after background correction using GraphPad Prism 8 at the emission wavelength of 475 nm. The experiment was repeated three times.

### Circular dichroism (CD) study

The far-UV circular dichroism spectra for the dilute and dense phase of proteins (with PEG-8000 10% w/v) immediately after LLPS (0 h) and after 48 h incubations were recorded using JASCO-1500 CD spectrophotometer (USA) in a 0.1 cm microcuvette (Hellma Forest Hills, NY). The samples were prepared by high-speed centrifugation, which resulted in the separation of two different phases (dilute phase monomeric protein and the dense liquid condensate)^49^. After removing most of the upper dilute phase, a small amount of the solution at the bottom of the tube was taken and diluted to 200 µl for CD measurements. Note, the dilution of the dense phase protein is unavoidable for CD due to very high dynode voltage and light scattering in the CD of the original dense phase suspension. The spectra were recorded for the wavelength range of 260-198 nm at 20 °C with a scanning speed of 200 nm/min. Three accumulations for each sample were acquired and the experiment was done in duplicate. The buffer subtraction and smoothing of the data were done as per the manufacturer’s instructions. The data was plotted using KaleidaGraph software.

### Fourier-transform infrared (FTIR) spectroscopy

FTIR spectroscopy was performed to determine the secondary structure of the proteins. All proteins which are purified from SEC were incubated at 37 °C for LLPS in the presence of 10% (w/v) PEG-8000. After phase separation, the dilute and dense phases of proteins were separated using high-speed centrifugation. The dilute and dense phases of the proteins without dilution were spotted on the KBr pellet and were subsequently dried under IR (infra-red) lamp. Vertex 80 FTIR system equipped with a DTGS detector (Bruker, Leipzig, Germany) was used to record the spectra in the range of 1800–1500 cm^−1^. Each spectrum was recorded using an average of 32 scans at a resolution of 4 cm^−1^. Fourier self-deconvolution (FSD) method was used to deconvolute the spectra corresponding to the wavenumbers 1700 − 1600 cm^−1^ ^125^. The Lorentzian curve fitting procedure was employed to fit the spectra using Opus-65 software (Bruker, Leipzig, Germany) as per the manufacturer’s instruction. The FTIR spectroscopy was also done after 48 h of phase separation for the selected proteins, which showed low FRAP recovery (β-cas, CATA, GG, LT, Tau, and α-Syn). The data was plotted using KaleidaGraph software. The experiments were performed twice with similar observations. The statistical significance was calculated using a two-tailed t-test (95% confidence interval) with *p*-values, *p* < 0.001, *p* < 0.002, *p* < 0.033, and *p* > 0.12 indicated by (***), (**), (*) and (ns), respectively.

### Protein sequence analysis and correlation plot parameters

The molecular weight, positively charged and aromatic residues of the proteins were determined using the Expasy ProtParam tool from the protein sequences obtained from Uniprot (Supplementary Table 1). Solvent-accessible surface area (SASA) was calculated using an existing SASA algorithm present in the VMD (Visual Molecular Dynamics)^66^ software package.

For determining the *C*_*sat*_ using the predictive model, we computed the solvent-accessible surface area (SASA) using available PDB structure files for the proteins. For IDPs, we used solved structure ensembles from Protein Ensemble Database (proteinensemble.org) and calculated the averaged SASA values from the structure ensembles. The structure of FUS (AlphaFold ID: AF-P35637-F1) and TDP43 (AlphaFold ID: AF-Q13148-F1) were obtained from the AlphaFold database whereas the structural information of p53 (PDB ID: 8F2H) and HSA (PDB ID: 4LB2) was obtained from the Protein data bank. The amino acids were categorized into four categories, namely aromatic (WFYH), polar (PTSNQ), charged (RKED), and hydrophobic (ACGILMV). The effective exposure of each residue (amino acid) was calculated as the ratio of the exposed surface area (from SASA calculations) to the maximum possible exposed surface area of each amino acid. The maximum solvent-accessible surface area for each amino acid was taken from the Tien et al.^126^. For each amino acid, the effective exposure will be in the range of 0 to 1, where 1 represents the amino acid being completely solvent accessible while 0 represents the amino acid is buried within the protein and is not solvent accessible. Then, we computed the net exposure for each category (aromatic, polar, charged, and hydrophobic) by summing each residue of the respective categories. Once we have the effective exposure of aromatic, polar, charged and hydrophobic residues for individual proteins, we fit these data using the following model.

6 $$\log ({{{{{{\rm{C}}}}}}}_{{{{{{\rm{sat}}}}}}})={{{{{\rm{A}}}}}}\times {{{{{{\rm{N}}}}}}}_{{{{{{\rm{Polar}}}}}}}+{{{{{\rm{B}}}}}}\times {{{{{{\rm{N}}}}}}}_{{{{{{\rm{Hydrophobic}}}}}}}+{{{{{\rm{C}}}}}}\times {{{{{{\rm{N}}}}}}}_{{{{{{\rm{Charge}}}}}}}+{{{{{\rm{D}}}}}}\times {{{{{{\rm{N}}}}}}}_{{{{{{\rm{Aromatic}}}}}}}+{{{{{\rm{E}}}}}}$$

Here each quantity (${N}_{{Polar}}$, ${N}_{{Hydrophobic}}$, ${N}_{{Charge}}$ and ${N}_{{Aromatic}}$) is defined as the summation of the effective exposure of those respective residues on the surface and *C*_*sat*_ is the saturation concentration at which the protein undergoes phase separation as obtained from experiments. To find the parameters A, B, C, D and E, we did multiple regression analysis to fit the model to the available protein data for proteins under this study (${{data\; is}:N}_{{Polar}}$, ${N}_{{Hydrophobic}}$, ${N}_{{Charge}}$, and ${N}_{{Aromatic}}$ and *C*_*sat*_). We used the scikit learn package in Python to do the multiple regression and found the 5 unknown variables (A, B, C, D and E). Thus, by supplying each quantity ${N}_{{Polar}}$, ${N}_{{Hydrophobic}}$, ${N}_{{Charge}}$, and ${N}_{{Aromatic}}$ from the structure of a protein, one can predict the approximate *C*_*sat*_ using the above equation.

All the graphs were plotted using the OriginPro 2021 (Origin Lab, USA) software.

### Surface plasmon resonance (SPR) analysis

The homotypic protein-protein interactions were determined using surface plasmon resonance (SPR) spectroscopy (BIAcore T200, GE Healthcare). SEC-isolated proteins were immobilized on the CM5 or CM3 sensor chip where immobilization levels of ~ 800-1000 response units were achieved for all proteins. The same protein with an increasing concentration range (GG: 25-500 nM, β-cas: 30 nM-2 μM, Chymo: 0.1-1 μM, CA: 5-80 μM, Mb: 10-60 μM, α-Syn: 5-100 μM and Ub: 25-250 μM) were injected into the microfluidic channel. To determine the effect of NaCl in GG, α-Syn, we immobilized the respective proteins in the sensor chip and allowed the same protein to pass through the chip in the presence of 150 mM salt. A similar experiment was done with Ub in the presence of 2 M urea. The contact time and dissociation time for the protein samples were set as follows: For GG (120 s and 360 s), β-cas (90 s and 400 s), Chymo (50 s and 120 s), CA (90 s and 300 s), Mb (45 s and 120 s), α-Syn (90 s and 300 s), Ub (60 s and 360 s). The contact time and flow rate for regeneration were set as follows: for GG (30 s and 40 μl/min), β-cas (60 s and 50 μl/min), Chymo (20 s and 20 μl/min), CA (30 s and 25μl/min), Ub (30 s and 35 μl/min). The response unit for the blank run (20 mM sodium phosphate buffer, pH 7.4) was used as a baseline and was subtracted from the response unit of the protein samples. The resultant response unit for the protein samples after the blank correction was fitted in the two-state model for all proteins except α-Syn and Chymo (one-state model, using SPR module). From the respectively fitted sensogram, the dissociation constant (K_D_) was determined using Biacore T200 software. The response unit curve was plotted with respect to time using OriginPro 2021 (Origin Lab, USA) software. The statistical significance was calculated using a two-tailed t-test (95% confidence interval) with *p*-values, *p* < 0.001, *p* < 0.002, *p* < 0.033, and *p* > 0.12 indicated by (***), (**), (*) and (ns), respectively.

### Reporting summary

Further information on research design is available in the Nature Portfolio Reporting Summary linked to this article.

### Supplementary information

Supplementary Information

Description of Additional Supplementary Files

Supplementary movie 1

Supplementary movie 2

Reporting Summary

### Source data

Source Data

## Data Availability

The authors declare that all the data supporting the findings of this study are available within the paper and in supplementary information files. All the data analysis was performed using published tools and packages and has been cited in the paper and supplementary information text. PDB (Protein Data Bank) IDs and PED (Protein Ensemble Database) IDs used in our study are available on the PDB and PED servers. PDB ID: 1B0L, 1QG5, 3V03, 1REX, 1FS3, 8F2H, 4LB2. PED ID: e001 (https://proteinensemble.org/entries/PED00017). Source data are provided with this paper.
