# Supplementary material for: Intermolecular interactions underlie protein/peptide phase separation irrespective of sequence and structure at crowded milieu
Source: Nat Commun. 2023 Oct 4;14:6199. doi: 10.1038/s41467-023-41864-9 (PMC10550955; doi:10.1038/s41467-023-41864-9)
Supplement: Supplementary file 1 — Supplementary Information [file 41467_2023_41864_MOESM1_ESM.pdf]

**SUPPLEMENTARY INFORMATION**

# Intermolecular interactions underlie protein/peptide phase separation irrespective of sequence and structure at crowded milieu

Manisha Poudyal[1,†], Komal Patel[1,2†], Laxmikant Gadhe[1], Ajay Singh Sawner[1], Pradeep Kadu[1], Debalina Datta[1], Semanti Mukherjee[1], Soumik Ray[1], Ambuja Navalkar[1], Siddhartha Maiti[1,3], Debdeep Chatterjee[1], Jyoti Devi[1], Riya Bera[1], Nitisha Gahlot[1], Jennifer Joseph[1], Ranjith Padinhateeri[1] and Samir K. Maji[1,2*]

[1]Department of Biosciences and Bioengineering, IIT Bombay, Powai, Mumbai-400076, India

[2]Sunita Sanghi Centre for Aging and Neurodegenerative Diseases, IIT Bombay, Powai, Mumbai 400076, India

[3]Current address: Department of Bioengineering, VIT Bhopal University, Bhopal-Indore Highway, Kothrikalan, Sehore, Madhya Pradesh-466114, India

[†]Contributed equally

*Correspondence: Prof. Samir K. Maji, Department of Biosciences and Bioengineering, IIT Bombay, Powai, Mumbai 400 076, India, Tel: + (91-22) 2576-7774, Fax: + (91-22) 2572 3480. Email: samirmaji@iitb.ac.in

**Contents** **Page no.**

1. Supplementary Figures (1-23) 3-26
2. Supplementary Table (1-4) 27-31
3. References 32

## Supplementary Figures

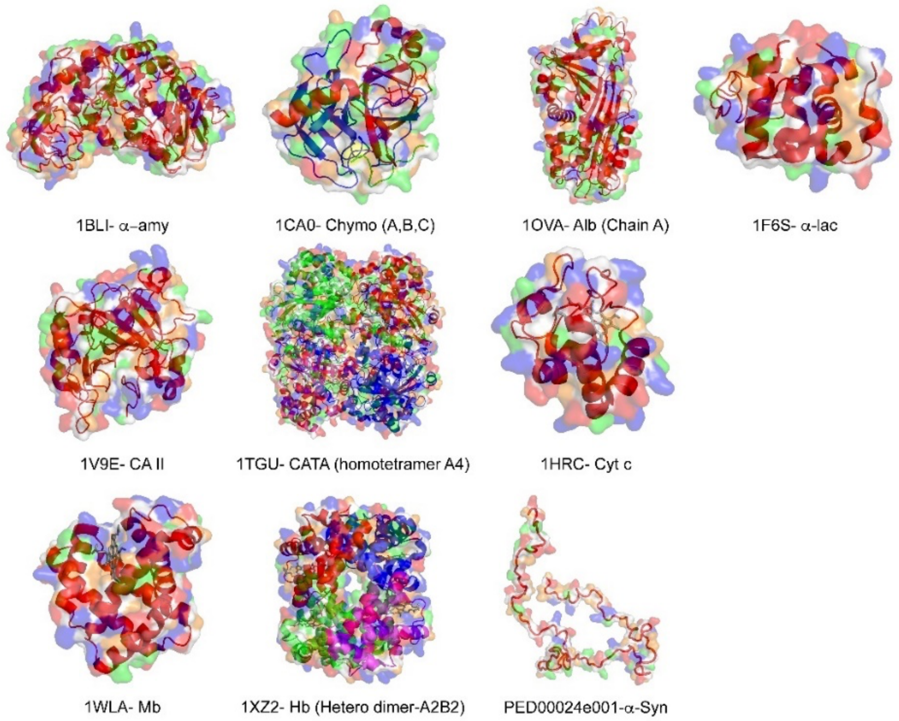


**Supplementary Figure 1. The structure along with the surface plot of various proteins.** The structure of all the proteins was generated from their PDB identification code (shown in respective image) and represented with the surface plot using PYMOL. The data shows that all proteins exhibit different secondary structures and surface charge distribution. The color representations are red, negatively charged residues; blue, positively charged residues and green, hydrophobic residues.

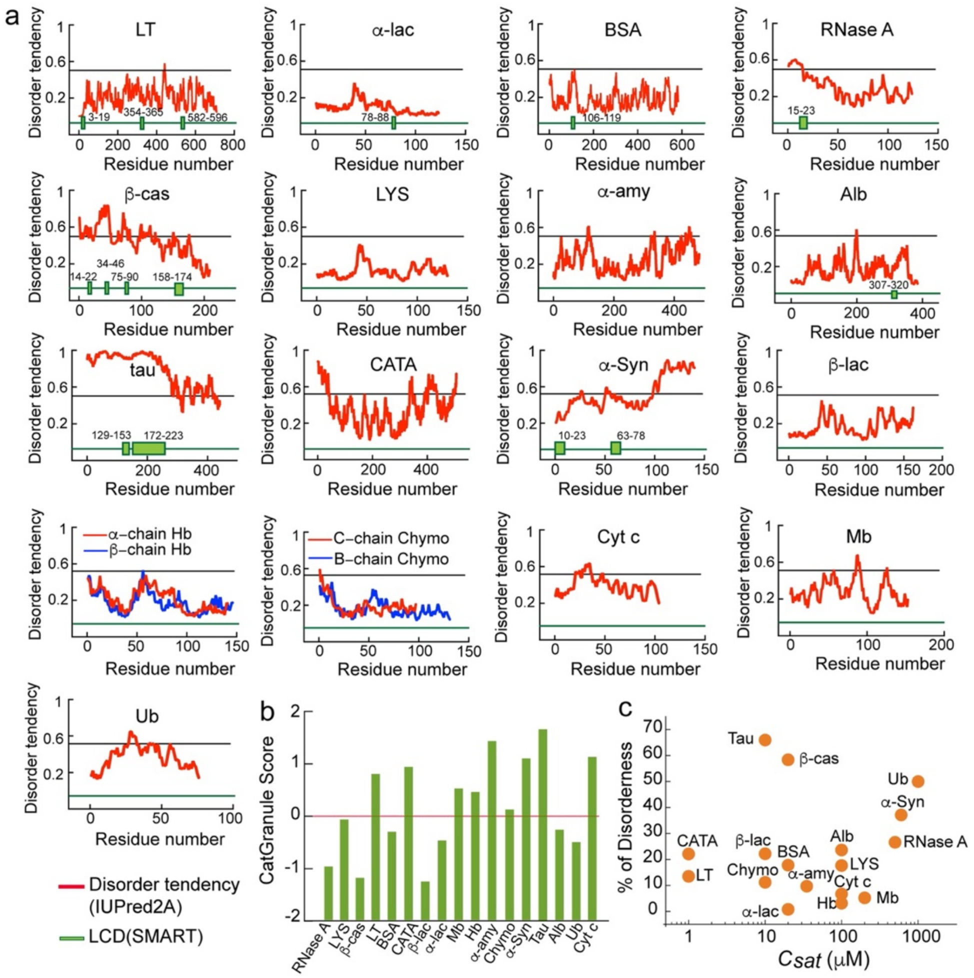


**Supplementary Figure 2. *In silico* analysis of the primary sequence of the various proteins.** *In silico* analysis of all the protein sequences showing (**a**) disorder tendency by IUPred2A[1], low complexity domains (LCDs) by SMART[2]. The disordered tendency is represented with red color where the threshold of 0.5 corresponds to the disordered region. The green color denotes the presence of LCDs. (**b**) The propensity for LLPS determined using CatGranule[3]. The line indicates the threshold value for LLPS. (**c**) The percentage of disorderness using PONDR[4] was plotted against $C_{sat}$ showing no correlation. Source data are provided as a Source Data file.

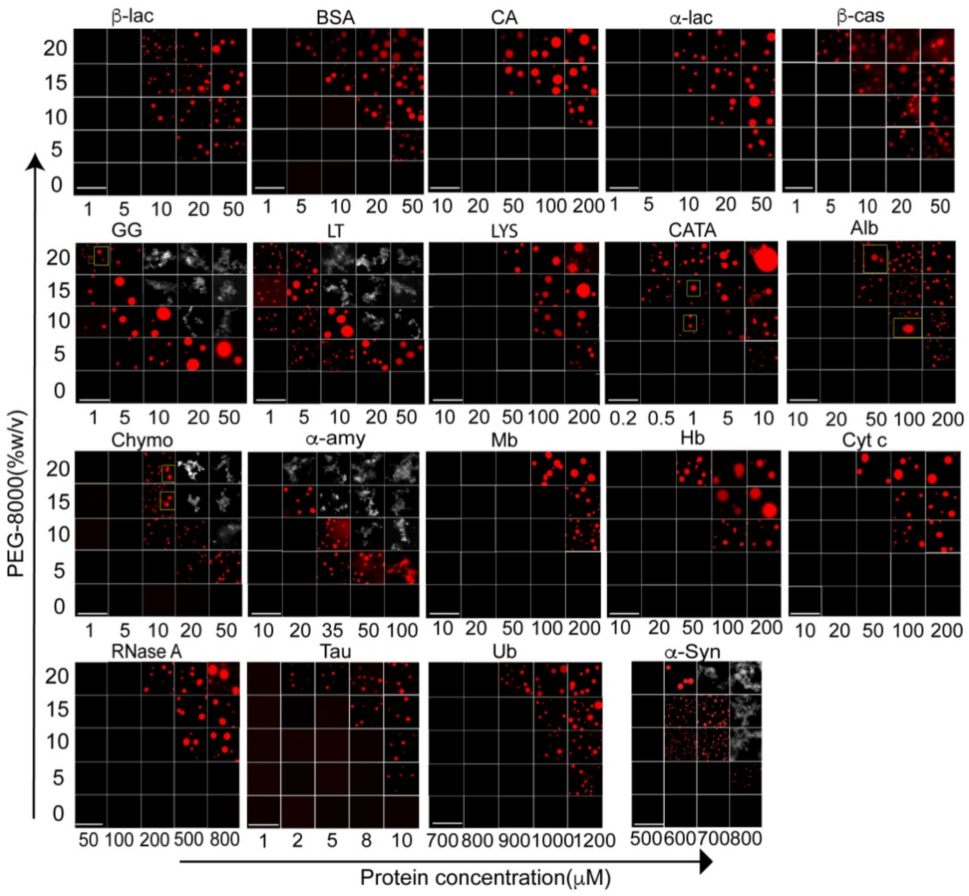


**Supplementary Figure 3. The phase separation regime of different proteins.** The phase separation regime of all NHS-Rhodamine labeled proteins [1:10 (v/v) of labeled versus unlabeled protein] at varying PEG-8000 concentrations (0%, 5%, 10%, 15% and 20% w/v) and protein concentrations in 20 mM sodium phosphate buffer at pH 7.4. The scale bar is 10 μm. The data showing low $C_{sat}$ for proteins such as LT and GG (1 μM) as well as very high $C_{sat}$ (≥500 μM) for proteins such as RNase A, Ub, and α-Syn. Representative images of precipitates are shown in 'greyscale' LUT. The experiment was performed three independent times with similar observations.

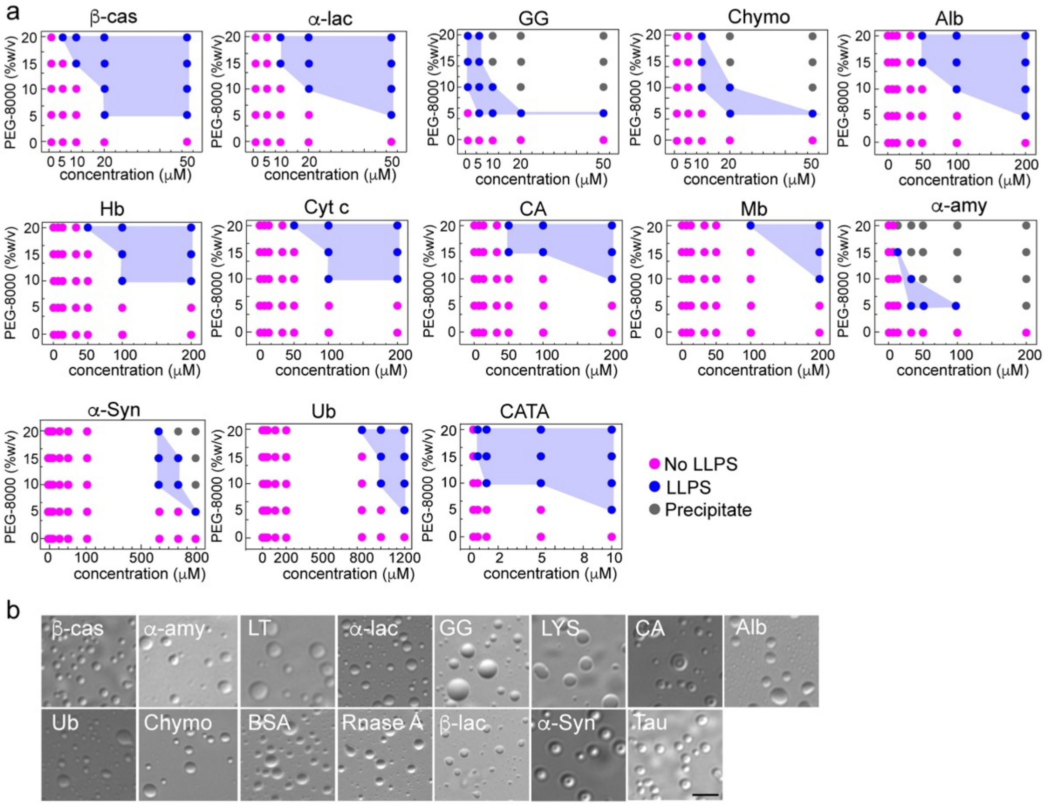


**Supplementary Figure 4. Phase diagram of different proteins.** (**a**) The schematic representation of the phase regime of all proteins depicting LLPS at varying protein and PEG-8000 concentrations (0%, 5%, 10%, 15% and 20% w/v) in 20 mM sodium phosphate buffer (pH 7.4). The different states are represented with various color codes. Pink color indicates no LLPS (soluble state), blue color indicated LLPS (condensate formation), and grey color indicates precipitates. The experiment was performed three independent times with similar observations. (**b**) Representative DIC images of condensate formation for different proteins during light scattering measurements (350 nm) at their $C_{sat}$ in the presence of 10% (w/v) PEG-8000 after nucleation. The scale bar is 5 μm. The experiment was performed two independent times with similar observations.

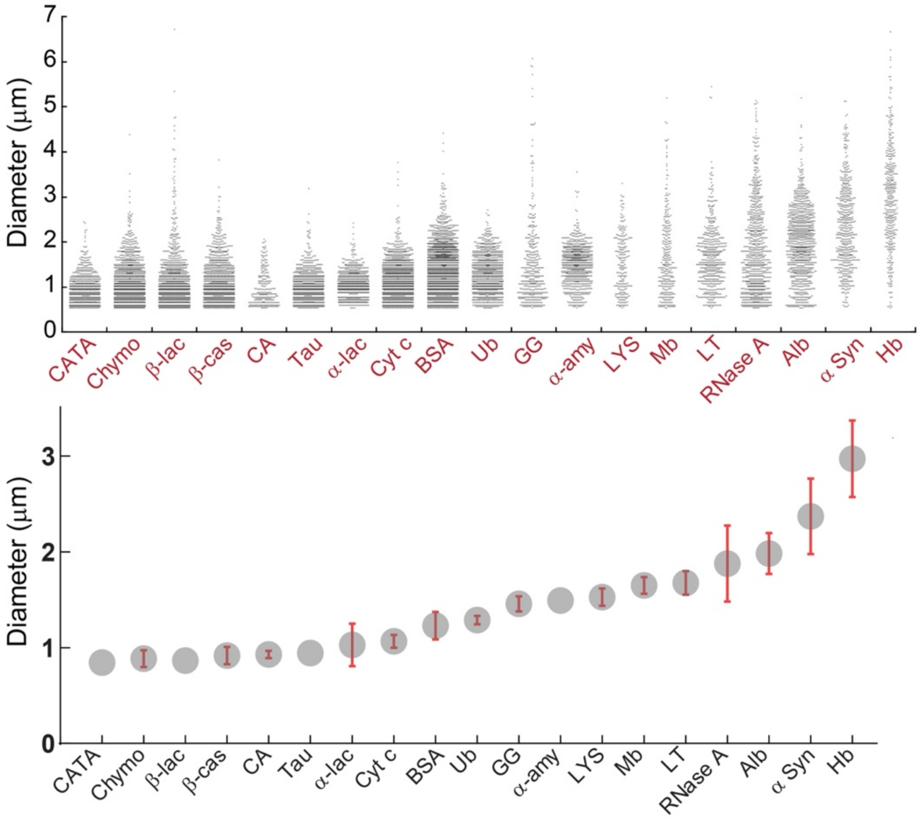


**Supplementary Figure 5. Size distributions and average diameter of protein condensates.** Size distribution (above) and average diameter calculation (below) show condensate size by various proteins. The data shows that a subset of proteins possess relatively lower-sized condensates. The data represent the mean ± s.d, for *n=3* independent experiments. Source data are provided as a Source Data file.

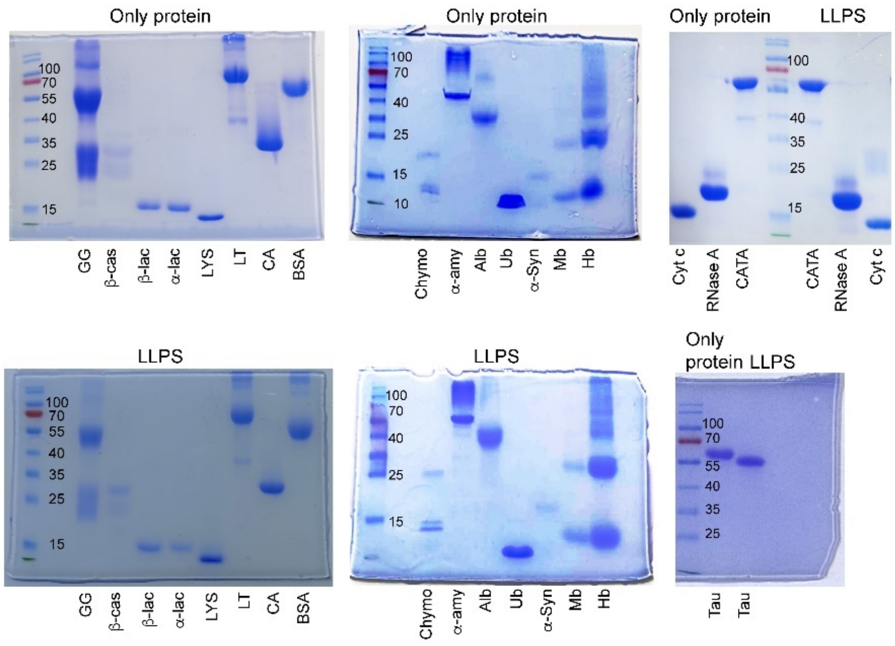


**Supplementary Figure 6. SDS-PAGE of SEC purified proteins.** The SDS-PAGE images showing the presence of protein bands at their respective molecular weights and confirming the purity of the protein with no degradation even after LLPS. Source data are provided as a Source Data file.

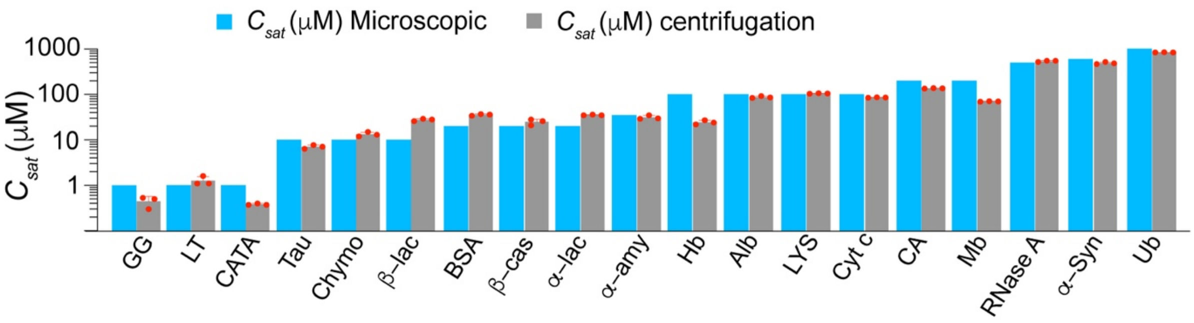


**Supplementary Figure 7**. **Determination of $C_{sat}$ of proteins**. Bar plot representing $C_{sat}$ of all proteins determined through microscopic observation (blue color) and centrifugation method (grey color). The dilute phase concentration of all proteins after phase separation determined through centrifugation signifies $C_{sat}$. The data represent the mean ± s.e.m. for $n=3$ independent experiments. $C_{sat}$ of most proteins determined through both methods show no significant difference. Source data are provided as a Source Data file.

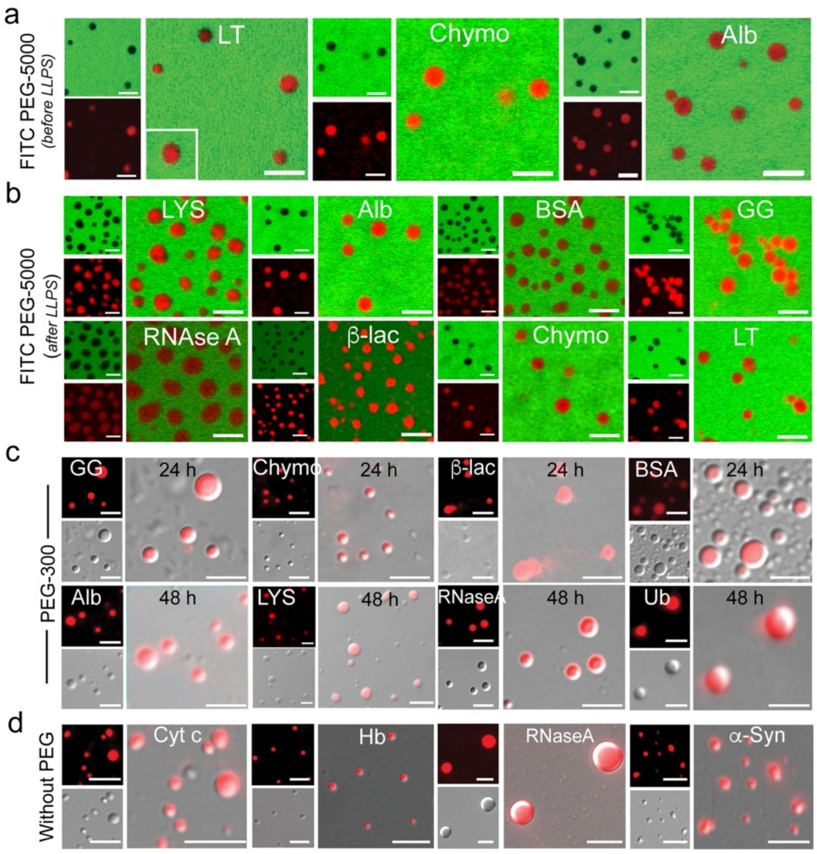


**Supplementary Figure 8**. **Liquid-liquid phase separation of proteins *in vitro* in the presence of FITC-labeled PEG-5000, PEG-300 and absence of PEG-8000. (a)** Representative confocal microscopy images of selected NHS-Rhodamine labeled proteins [10% (v/v) labeled to unlabeled] (LT, Chymo and Alb) in the presence of 10% PEG (w/v) (5% FITC-labeled PEG-5000 + 5% PEG-8000) showing LLPS with no PEG sequestration inside the condensates. All the samples were prepared under identical conditions using 20 mM sodium phosphate buffer (pH 7.4) and FITC-labeled PEG-5000 was added before the proteins undergo liquid-liquid phase separation. The scale bar is 5 µm. (**b**) Representative confocal microscopy images of selected NHS-Rhodamine labeled proteins [10% (v/v) labeled to unlabeled] showing LLPS in the presence of 10% PEG-8000 (w/v). Immediately after phase separation, 5% (w/v) FITC-labeled PEG-5000 was added and the confocal microscopy data showed no PEG sequestration inside the condensate. The scale bar is 5 µm. **(c)** Representative fluorescence microscopy images, DIC microscopy and DIC/fluorescence merged images showing phase separation of NHS-Rhodamine labeled proteins (GG, Chymo, β-lac, BSA, Alb, LYS, RNase A and Ub) in the presence of 10% (v/v) PEG-300. The time mentioned in each merged image indicating the time required for condensate formation. All the samples were prepared in 20 mM sodium phosphate buffer at pH 7.4. The scale bar is 5 µm. **(d)** Representative fluorescence, DIC microscopy and DIC/fluorescence merged images of a subset of NHS-Rhodamine labeled protein [1:10 (v/v) labeled to unlabeled protein] condensates formed in the absence of PEG-8000. The conditions required for phase separation are listed in Supplementary Table 3. The scale bar is 5 µm. All the experiments were performed three independent times with similar observations.

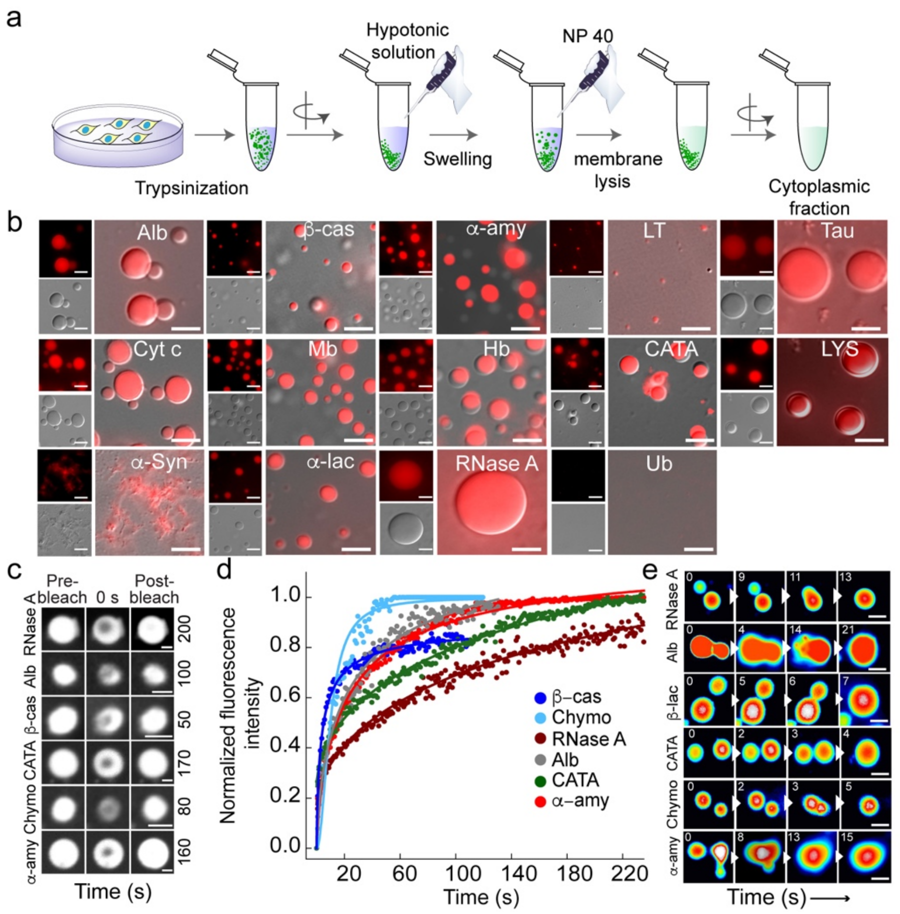


**Supplementary Figure 9. Liquid condensate formation in cellular lysate. (a)** Schematic representation depicting extraction procedure of cytoplasmic fraction from HeLa cell line. **(b)** Representative fluorescence, DIC microscopy and DIC/fluorescence merged images of the NHS-Rhodamine labeled [1:10 (v/v) labeled to unlabeled protein] condensates at their respective $C_{sat}$ in the presence of cellular lysate. The scale bar is 5 µm. The experiment was performed three independent times with similar observations. (**c**) Representative images showing selected protein condensates (RNase A, Alb, β-cas, CATA, Chymo and α-amy) during FRAP analysis [before bleaching, at bleaching (0 s), and after bleaching (respective recovery time shown at the right side in second)]. The images are represented in 'grey' LUT for better visualization. The scale bar is 5 µm. (**d**) Normalized FRAP (in arbitrary units) curves for the selected protein condensates (RNase A, Alb, β-cas, CATA, Chymo and α-amy) are plotted against time. *n=3* independent experiments were performed. (**e**) Time-lapse images showing fusion events of condensates formed by selected proteins over time (RNase A, Alb, β-cas, CATA, Chymo and α-amy) in the cytoplasmic extract. Images are represented in 'royal' LUT for better visualization. Representative results are shown. The time of fusion events are shown in respective images. The scale bar is 5 µm. The experiment was performed two independent times with similar observations. Source data are provided as a Source Data file.

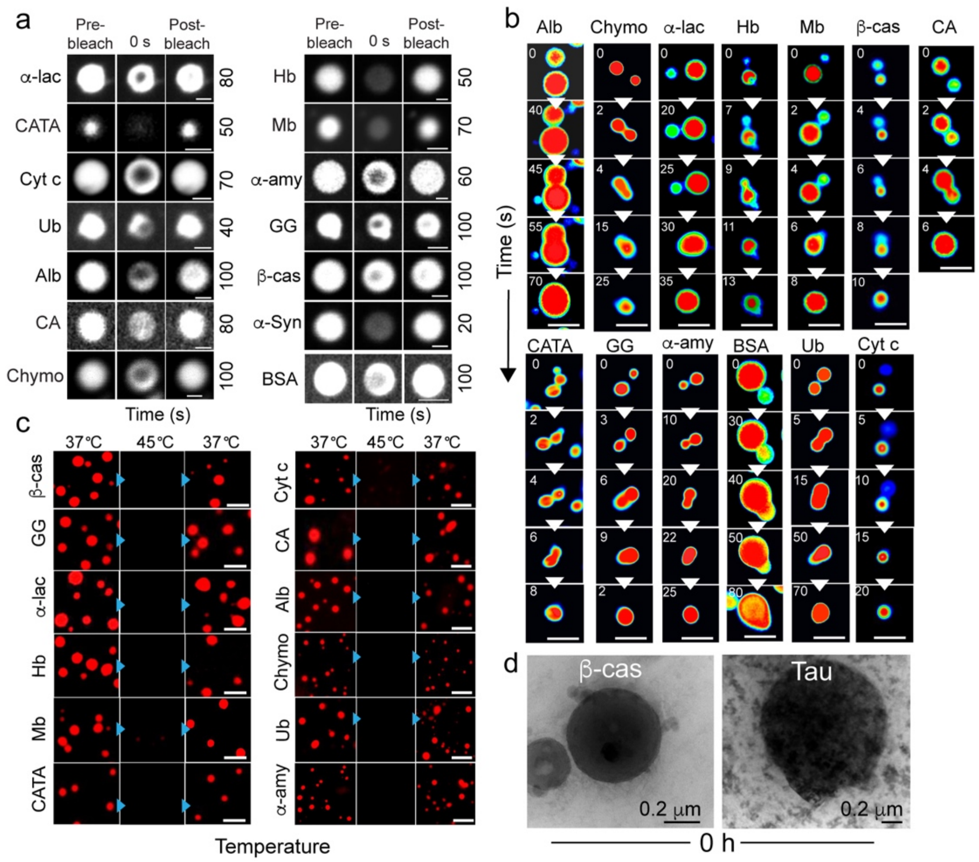

**Supplementary Figure 10. Liquid-like nature of different protein condensates.** (**a**) Representative image showing the different protein condensates immediately after LLPS (0 h) during FRAP analysis [before bleaching, at bleaching (0 s), and after bleaching (respective recovery time shown at the right side in second]. The images are represented in 'grey' LUT for better visualization. The experiment was performed three independent times with similar observations. The scale bar is 2 µm. (**b**) Representative time-lapse images of the proteins showing the fusion of small condensates upon contact, resulting in the formation of larger condensates over time. 'Royal' LUT was used for better visualization. The time for fusion events are shown in respective images. The experiment was performed two independent times with similar observations. The scale bar is 5 µm. (**c**) Thermo-reversibility of liquid condensates. The fluorescence microscopy images of the NHS-Rhodamine labeled [1:10 (v/v) labeled to unlabeled protein] condensates of different proteins at their $C_{sat}$ in the presence of PEG-8000 (10% w/v) indicating thermo-reversibility (37 °C → 45 °C → 37 °C). The experiment was performed two times with similar observations. The scale bar is 5 µm. (**d**) Representative TEM images of β-cas and Tau showing the morphology of protein condensate at LLPS (0 h). *n=2* independent experiments were performed.

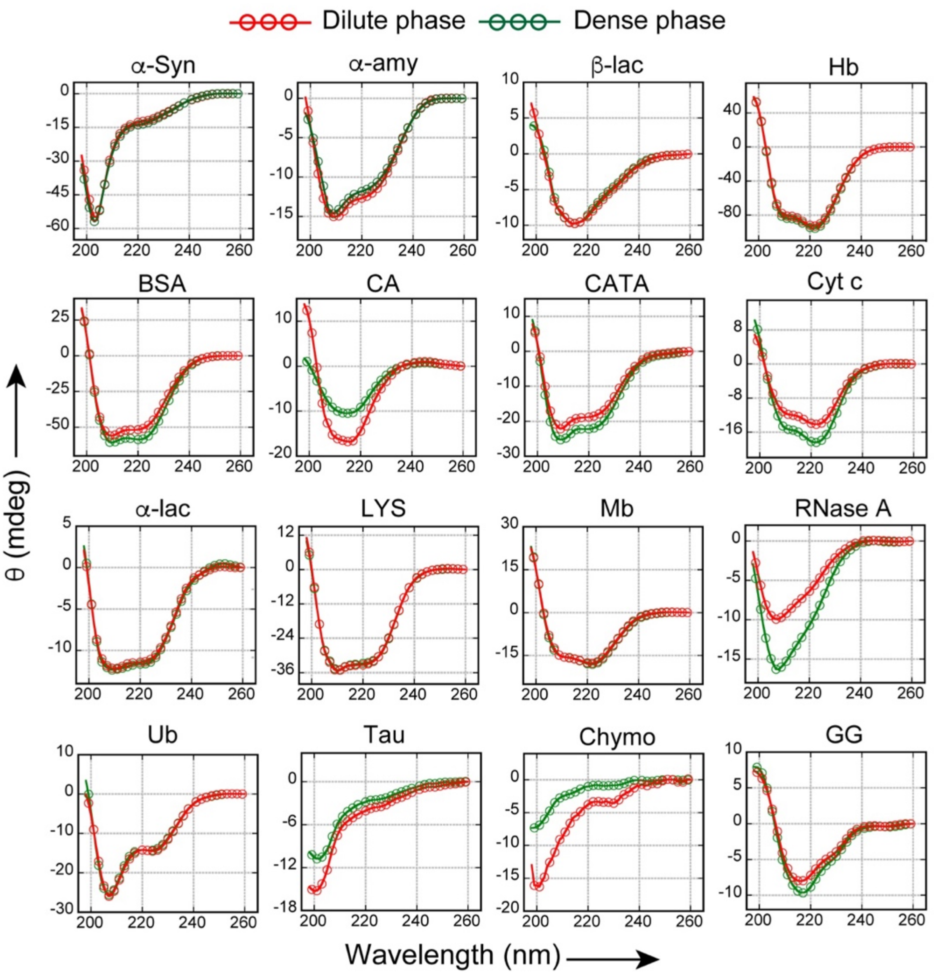


**Supplementary Figure 11. Secondary structural analysis of dense and dilute phases of various proteins.** The CD spectroscopic analysis of the dense and dilute phase of all proteins separated through centrifugation at 0 h showing that most of the proteins did not undergo large secondary structural transition due to LLPS. The red color represents the spectra of the dilute phase of protein in the presence of PEG-8000 (10% w/v) and green indicates CD spectra of the dense phase of protein in 20 mM sodium phosphate buffer (pH 7.4) in the presence of 10% (w/v) PEG-8000. The experiment was performed two independent times with similar observations. Source data are provided as a Source Data file.

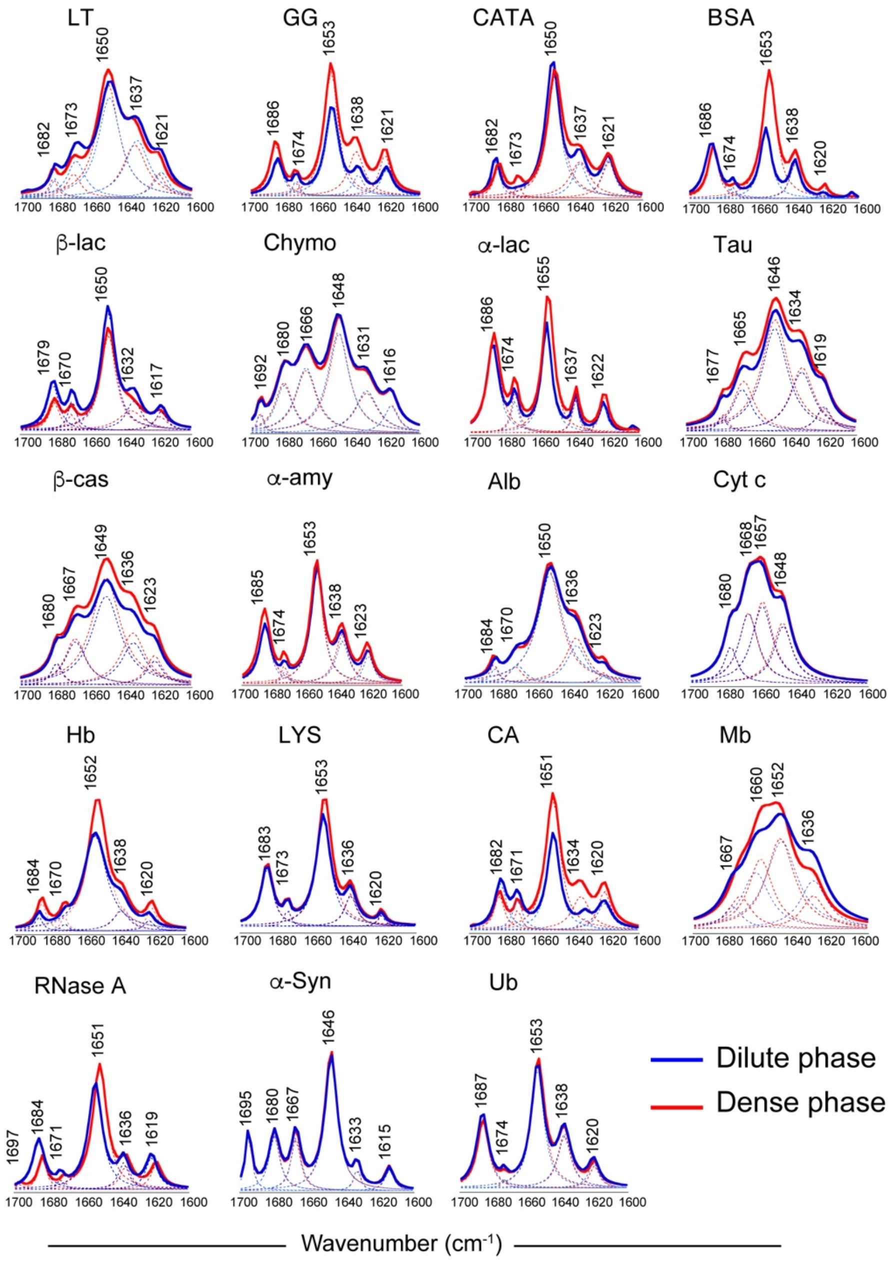


**Supplementary Figure 12. Determination of the secondary structure after phase separation (0 h) of the dilute and dense phase of the proteins using FTIR spectroscopy.** Deconvoluted FTIR spectra of the dilute (blue) and dense (red) phase of proteins showing the different secondary structures of protein after LLPS (0 h). The experiment was performed two times with similar observations. Source data are provided as a Source Data file.

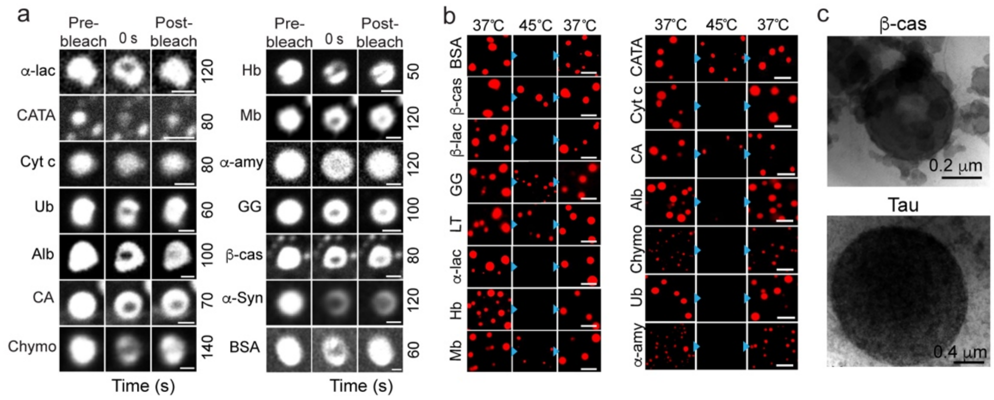


**Supplementary Figure 13. The viscoelastic transition of various proteins.** (**a**) Representative image showing the liquid condensates at 48 h during FRAP analysis [before bleaching, at bleaching (0 s), and after bleaching (respective recovery time shown at the right side in second)]. The data suggests the viscoelastic transition of the various protein condensates. The images are represented in 'grey' LUT for better visualization. The experiment was performed three times with similar observations. The scale bar is 2 µm. (**b**) Thermo-reversibility of protein condensates. The fluorescence microscopy images of the NHS-Rhodamine labeled [1:10 (v/v) labeled to unlabeled protein] condensates at their $C_{sat}$ after 48 h of incubation in the presence of PEG-8000 (10% w/v) indicating a lack of thermo-reversibility (37 °C → 45 °C →37 °C) after ageing (48 h) for a subset of protein condensates. The experiment was performed two times with similar observations. The scale bar is 5 µm. (**c**) Representative TEM images of β-cas and Tau showing the morphology of protein condensate at LLPS (48 h). *n=2* independent experiments were performed.

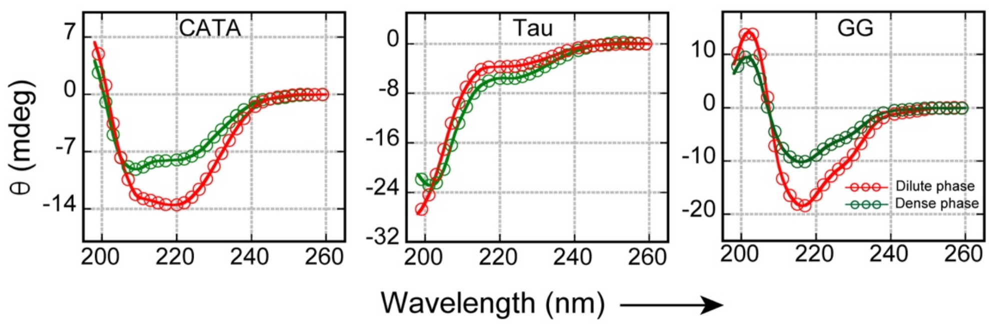


**Supplementary Figure 14. Secondary structural analysis of various proteins after 48 h of LLPS.** The CD spectroscopic analysis of dense and dilute phases of proteins (which showed substantial rigidification using FRAP data) after 48 h of LLPS. The red color represents the CD spectra of the dilute phase of the protein and the green represents the CD spectra of the dense phase of the protein after 48 h of incubation. Protein LLPS was done in 20 mM sodium phosphate buffer (pH 7.4) in the presence of 10% (w/v) PEG-8000. The experiment was performed two independent times with similar observations. Source data are provided as a Source Data file.

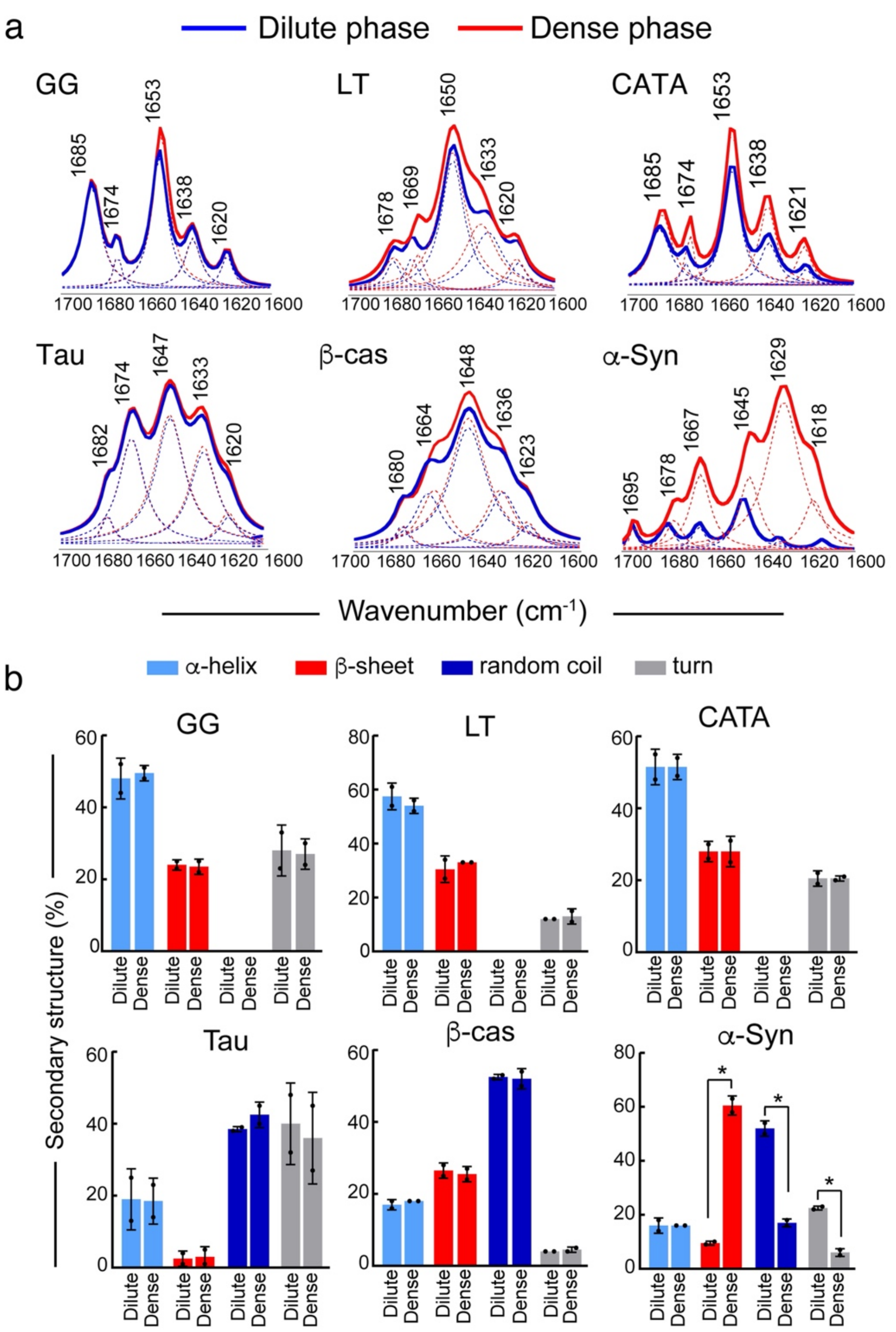


**Supplementary Figure 15. Determination of the secondary structure after phase separation (48 h) of the dilute and dense phase of the proteins using FTIR spectroscopy.** (**a**) The deconvoluted FTIR spectra of the dilute (blue) and dense (red) phase of proteins (GG, LT, CATA, Tau, β-cas and α-Syn) after LLPS (48 h) showing a significant structural change for α-Syn. (**b**) The deconvoluted FTIR spectra were used for calculating the percentage of secondary structure. The data values represent the mean ± s.d. for *n=2* independent experiments. The statistical significance was calculated using a two-tailed t-test (95% confidence interval) with no adjustments (*p*-values, $p < 0.001$, $p < 0.002$, $p < 0.033$, and $p > 0.12$ indicated by (***), (**), (*) and (ns), respectively). The p values of β-sheet dense Vs dilute is 0.03, random coil dense Vs dilute is 0.01 and turn dense Vs dilute is 0.01. Source data are provided as a Source Data file.

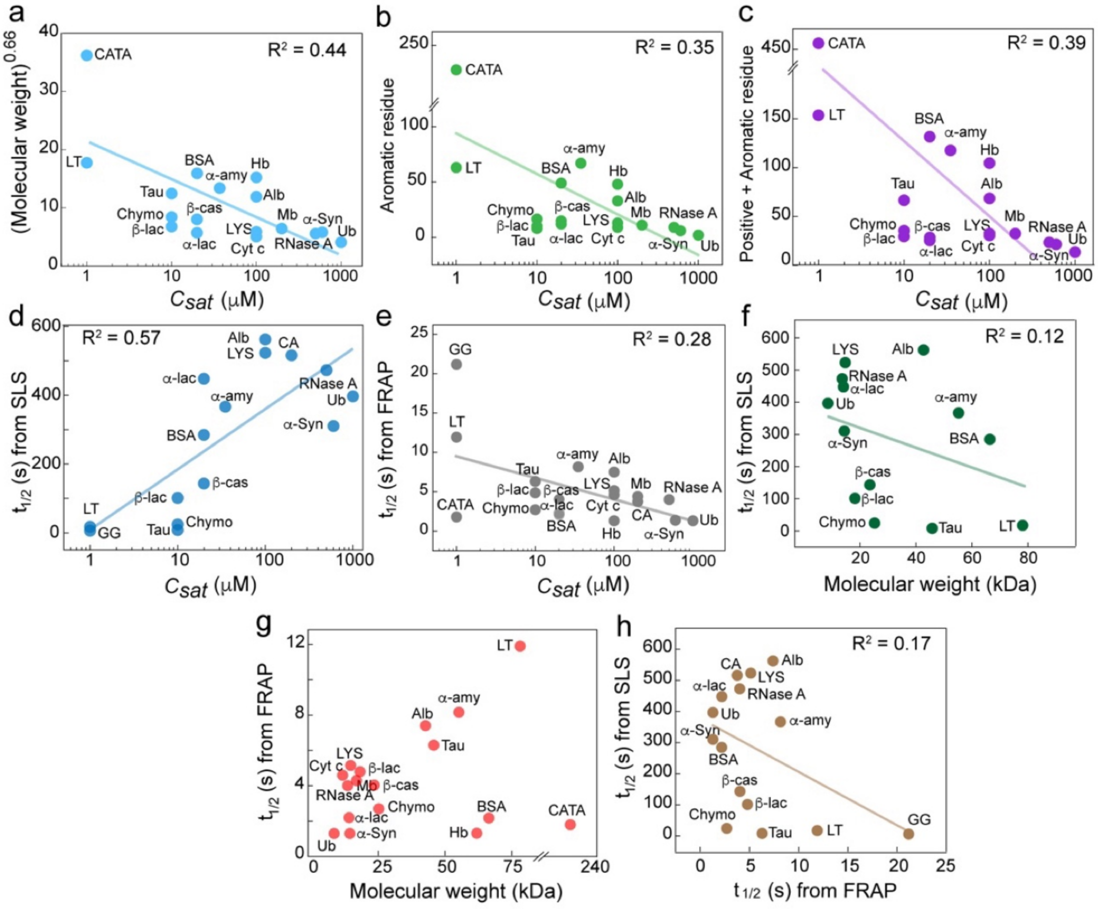


**Supplementary Figure 16. Correlation plots of protein-specific parameters. (a-e)** Correlation plots of (**a**) (molecular weight)$^{0.66}$ and $C_{sat}$ (**b**) number of aromatic residues and $C_{sat}$ (**c**) number of positive and aromatic residues (a measure of cation-π interactions) and $C_{sat}$. (**d**) $t_{½}$ calculated from the condensate growth curve (Fig. 1e) from static light scattering (SLS) experiments and $C_{sat}$. (**e**) $t_{½}$ from FRAP experiment and $C_{sat}$ showing no apparent correlation. (**f, g**) Correlation plots between $t_{½}$ from SLS and $t_{½}$ from FRAP with molecular weight of proteins exhibiting no correlation. (**h**) Correlation plot of $t_{½}$ from SLS and $t_{½}$ from FRAP. All these plots show no apparent correlation suggesting that the driving forces are complex and diverse, which could not be easily extrapolated at present. Source data are provided as a Source Data file.

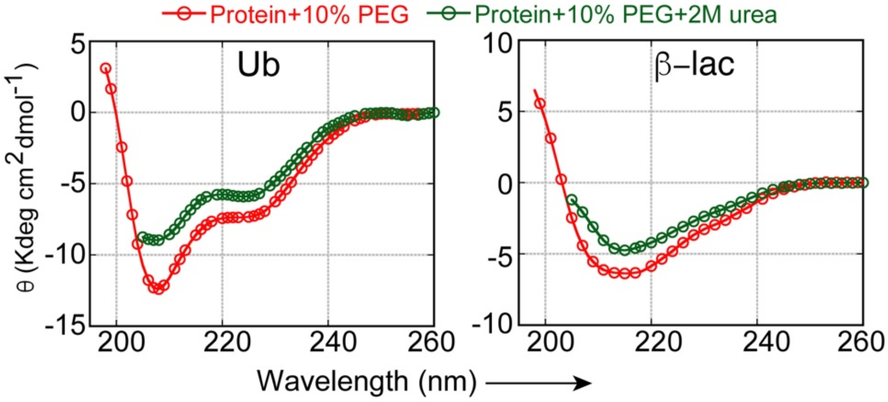


**Supplementary Figure 17. CD spectra of Ub and β-lac in the presence and absence of 2 M urea.** The CD spectroscopic analysis of Ub and β-lac in the presence (green) and absence (red) of 2 M urea in the presence of 10% PEG-8000 (w/v). The figure showing no significant spectral change upon the addition of 2 M urea for Ub and β-lac. The experiment was performed two independent times with similar observations. Source data are provided as a Source Data file.

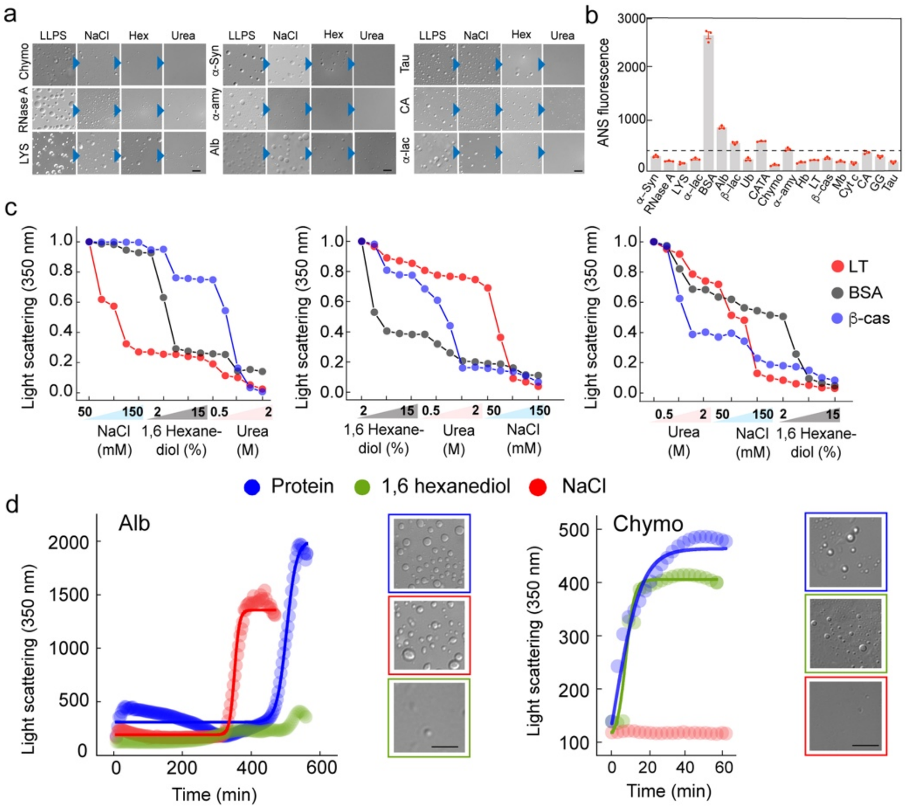

**Supplementary Figure 18. Determination of intermolecular interactions responsible for protein LLPS. (a)** Representative DIC images showing the presence and absence of condensates in the presence of various additives (150 mM NaCl, 15% 1,6 hexanediol and 2 M Urea) during the light scattering experiment. The experiment was performed two times with similar observations. Scale bar is 5 µm. (**b**) ANS fluorescence (in arbitrary units) of all proteins showing the extent of exposed hydrophobic surface. The red dots represent the data points from three independent experiments. The data represent the mean ± s.e.m. for $n=3$ independent experiments. (**c**) Normalized static light scattering at 350 nm of LT (red), BSA (black) and β-cas (blue) showing a decrease in light scattering value by the titration of different concentrations of additives in an altered manner on pre-formed protein condensates. (**d**) Inhibition of protein condensate in the presence and absence of NaCl (150 mM) and 10% (w/v) 1,6-hexanediol to disrupt electrostatic and hydrophobic interactions, respectively. The static light scattering measurement at 350 nm of Alb and Chymo at their respective $C_{sat}$ in the presence of 10% (w/v) PEG-8000 shows scattering due to LLPS. The blue color indicates spectra of protein in the presence of 10% (w/v) PEG-8000. Green and red colors denote the light scattering measurement of respective proteins in the presence of 1,6-hexanediol (10%) and NaCl (150 mM), respectively. Representative DIC images showing the absence or presence of condensate formation in the presence of various additives. The scale bar is 5 µm, $n=2$, independent experiments. Source data are provided as a Source Data file.

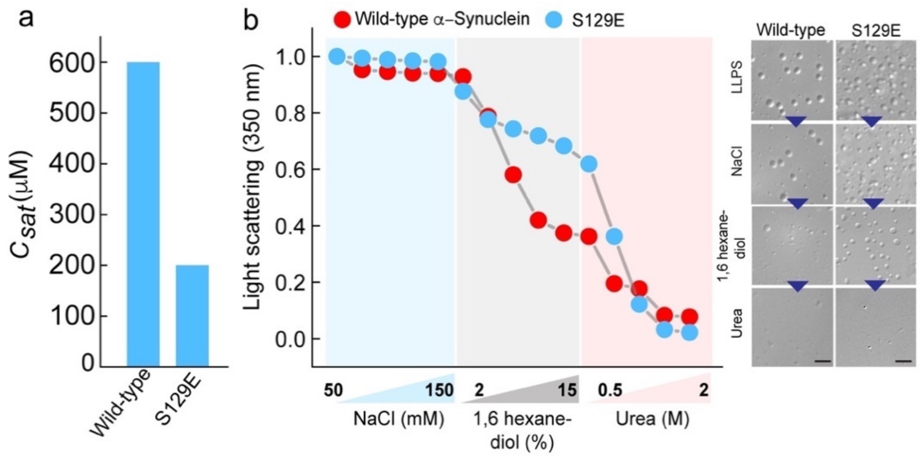


**Supplementary Figure 19. LLPS of α-Syn phosphomimetic mutant S129E *in vitro*: (a)** Bar graph showing the $C_{sat}$ of wild-type α-Syn and the phosphomimetic mutant, S129E in the presence of 10% PEG-8000 (w/v) in 20 mM sodium phosphate buffer (pH 7.4). The data shows wild-type α-Syn requires a very high saturation concentration (600 μM) for condensate formation whereas, S129E requires a lower concentration (200 μM) for phase separation. The experiment was performed three times with similar observations. **(b)** Normalized static light scattering measurement at 350 nm showing a decrease in the scattering values upon addition of different concentrations of additives (NaCl, 1,6 hexanediol and Urea) on preformed condensates of S129E and wild-type α-Syn *(Left)*. Representative DIC microscopy images confirming the presence/absence of protein condensates after the addition of additives during the light scattering measurement *(Right)*. The scale bar is 5 μm, *n=2*, independent experiments. Source data are provided as a Source Data file.

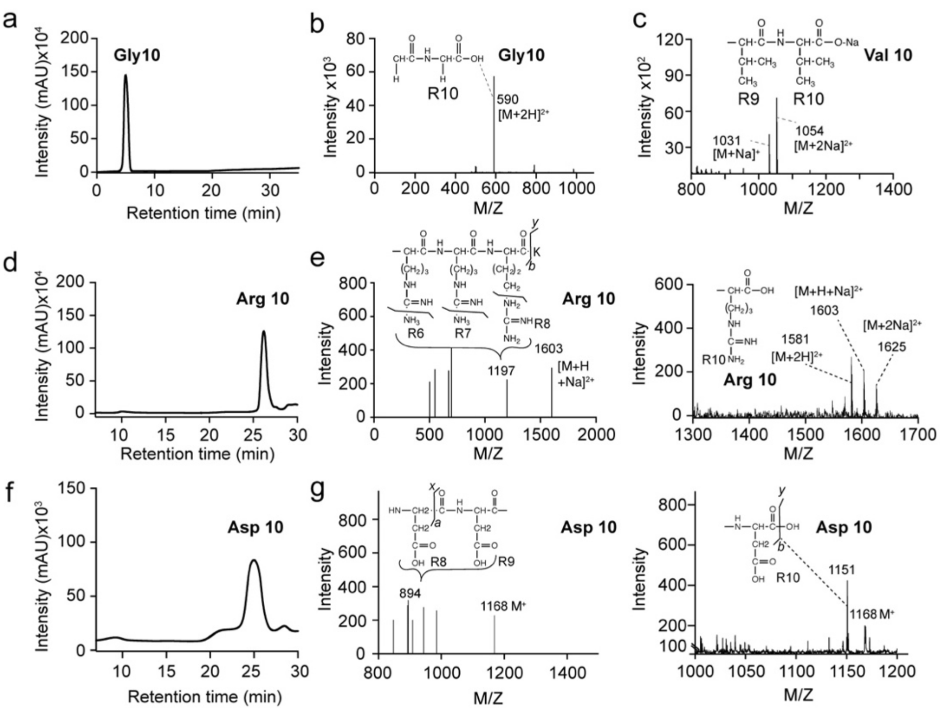


**Supplementary Figure 20. Purity and characterization of polypeptides.** (**a**) HPLC chromatogram (in milli absorbance unit) of $(Gly)_{10}$ peptide with a retention time of ~ 5 min. (**b**) MALDI profile of $(Gly)_{10}$ showing molecular weight peak at m/z=590 $[M+2H]^{+2}$. (**c**) MALDI profile of $(Val)_{10}$ showing molecular ion peaks associated with sodium at m/z=1031 $[M+Na]^{+}$ and m/z=1054 $[M+2Na]^{2+}$. (**d**) HPLC chromatogram (in milli absorbance unit) of $(Arg)_{10}$ showing a retention time of ~ 27 min. (**e**) The ESI-MS (left) and MALDI profile (right) of $(Arg)_{10}$ showing a common molecular ion peak at m/z=1603 $[M+H+Na]^{2+}$. The ESI-MS peak at m/z=1197 is a combined molecular ion peak of multiple fragmentation of side chain residues as represented in inset structure. The MALDI profile showed other molecular ion peaks at m/z=1581, 1625 representing the association of $[M+2H]^{2+}$ and $[M+2Na]^{2+}$, respectively. (**f**) HPLC chromatogram (in milli absorbance unit) of $(Asp)_{10}$ showing a retention time of ~25 min. (**g**) The ESI-MS (left) and MALDI profile (right) of $(Asp)_{10}$ showing molecular ion peak at m/z=1168 $[M]^{+}$. The fragmentation peak in ESI-MS at m/z=894 Da is represented in the inset structure, whereas the MALDI profile showed a peak at m/z=1151 Da after the fragmentation of the hydroxyl group from the C-terminus. Source data are provided as a Source Data file.

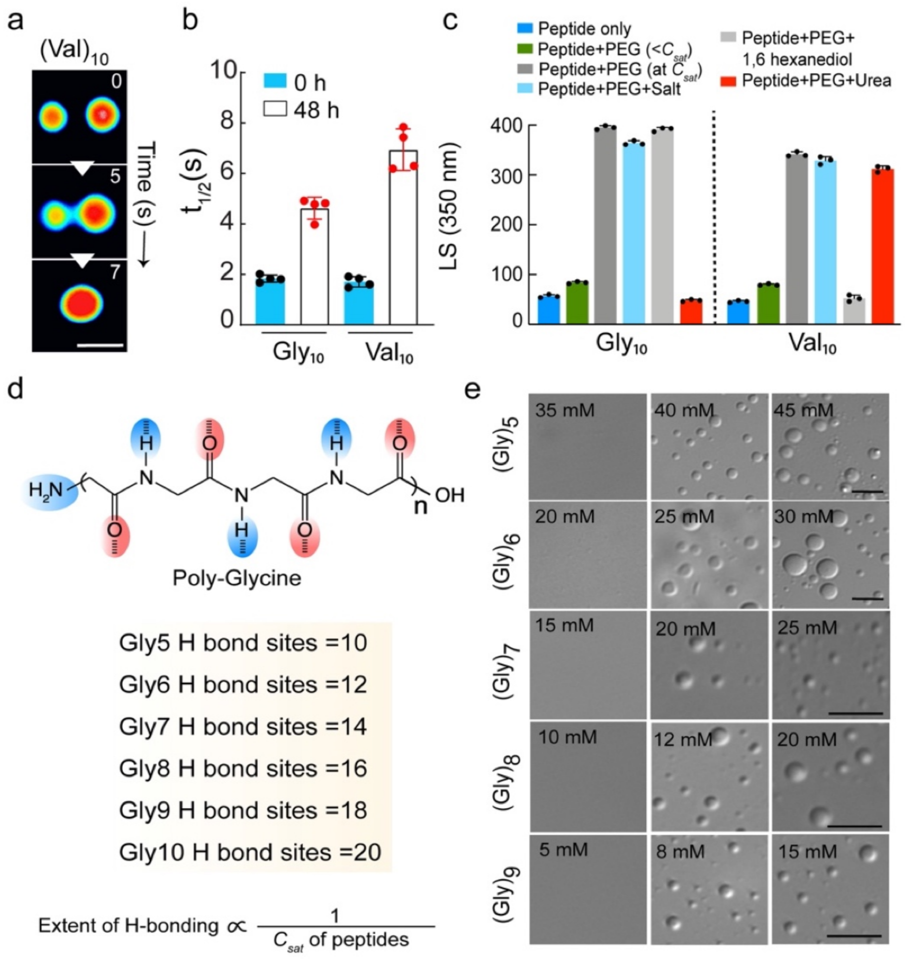


**Supplementary Figure 21. Characterization of poly-Gly and poly-Val condensates. (a)** Time-lapse images of $(Val)_{10}$ condensate depicting the fusion of condensates and subsequent growth over time. Represented in 'royal' LUT for better visualization. The time (in second) for fusion events are shown in respective images. The experiment was performed two times with similar observations The scale bar is 5 µm. **(b)** The bar plot showing $t_{1/2}$ values of fluorescence recovery after photobleaching of $(Gly)_{10}$ and $(Val)_{10}$ condensates at 0 h (blue) and 48 h (white). The data represent the mean ± s.e.m. for *n=3* independent experiments. **(c)** Static light scattering measurements at 350 nm of $(Gly)_{10}$ and $(Val)_{10}$ in the presence of different additives showing the effect of these additives for condensate formation. The experiment was performed three times with similar observations **(d)** Schematic representation showing the total number of possible H-bonding sites (donor + acceptor sites) of different Gly polypeptides. **(e)** DIC images of different Gly polypeptides at increasing peptide concentrations in the presence of 10% (w/v) PEG-8000 showing $C_{sat}$ of peptide required for condensates formation. The scale bar is 5 µm, *n=2* independent experiments. Source data are provided as a Source Data file.

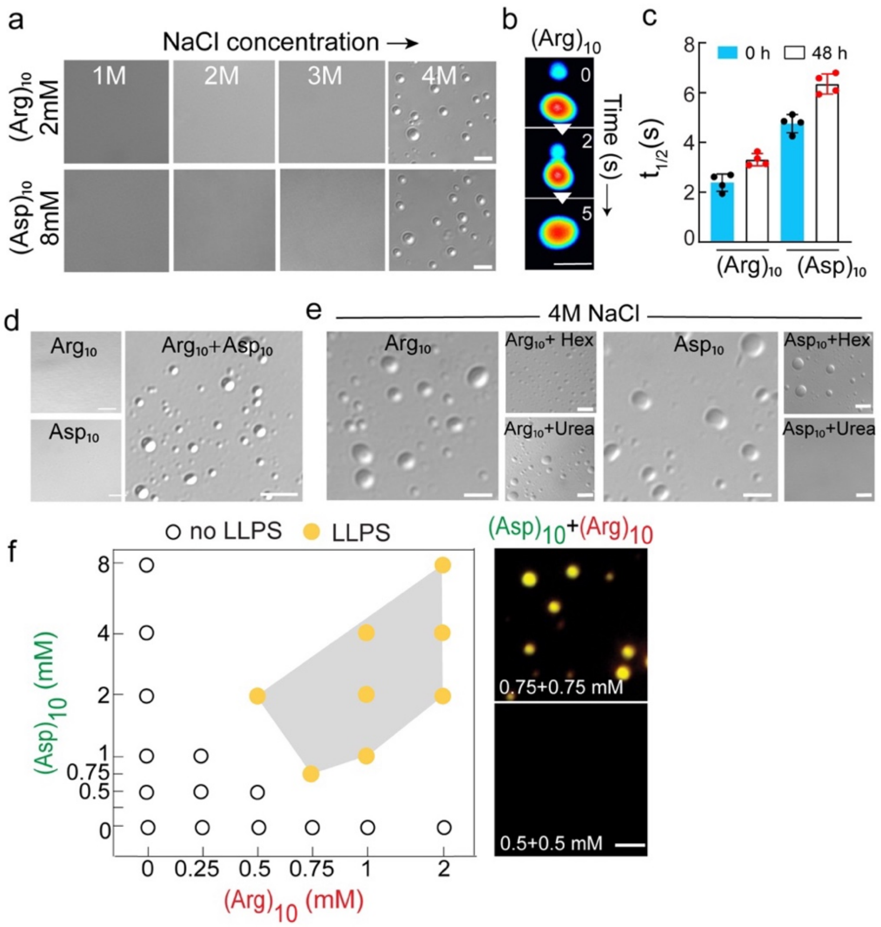


**Supplementary Figure 22. LLPS of charged homo polypeptides**. **(a)** DIC image showing minimum NaCl concentration required for condensate formation by $(Arg)_{10}$ and $(Asp)_{10}$ at their respective $C_{sat.}$ The experiment was performed three times with similar observations. The scale bar is 5 µm. **(b)** Time-lapse images of $(Arg)_{10}$ condensate depicting the fusion of two condensates into one large-sized condensate over time. Represented in 'royal' LUT for better visualization. The time for fusion events (in second) is shown in respective images. The experiment was performed two times with similar observations. The scale bar is 5 µm. **(c)** The bar plot showing $t_{1/2}$ values of fluorescence recovery after photobleaching of $(Arg)_{10}$ and $(Asp)_{10}$ condensates at 0 h (blue) and 48 h (white). The data represent the mean ± s.e.m. for *n=3* independent experiments. **(d)** DIC microscopy images showing condensate formation by $(Arg)_{10}$ and $(Asp)_{10}$ when mixed (in the absence of salt) at their respective $C_{sat}$. Respective polypeptide alone at $C_{sat}$ without mixing (in the absence of salt) are used as controls (left). The scale bar is 5 µm. The experiment was repeated three times with similar observations. **(e)** Representative DIC images of $(Arg)_{10}$ and $(Asp)_{10}$ condensates showing the effect of 1,6 hexanediol and urea for phase separation. Individual polypeptides at $C_{sat}$ in the presence of salt are used as control (left, in the absence of any additives). The experiment was performed two times with similar observations **(f)** The multicomponent phase separation by $(Asp)_{10}$ and $(Arg)_{10}$ depicting the phase regime at varying peptide concentrations ratio in the presence of PEG-8000 in 20 mM sodium phosphate buffer (pH 7.4). An open black circle indicates no LLPS and yellow color indicates co-LLPS (condensate formation). Representative

fluorescence images (right) showing multicomponent condensate formation by $(Asp)_{10}$ (FITC labeled) and $(Arg)_{10}$ (NHS-Rhodamine labeled) in the presence of 10% (w/v) PEG-8000 when mixed in a specific ratio. The experiment was repeated three times with similar observations. The scale bar is 5 μm. Source data are provided as a Source Data file.

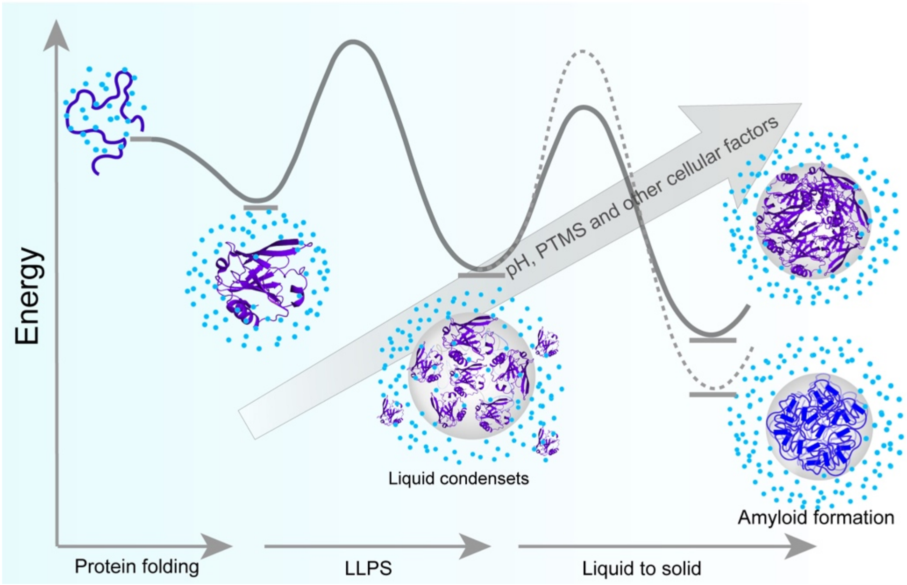


**Supplementary Figure 23. Liquid-liquid phase separation and viscoelastic transition by proteins.** The schematic represents how folded proteins might undergo LLPS and viscoelastic transition. Blue dots represent water molecules (either surface-bound or free in solution). Rigidification of condensates can result due to crystal packing or amorphous aggregation. Some proteins can also undergo rigidification due to amyloid aggregation. The diagram indicates different protein states and the corresponding energy barrier for their interconversion. The LLPS and condensate rigidification of proteins can be further modulated by various cellular factors including post-translational modifications of proteins.

**Supplementary Table 1. Various proteins used for the LLPS study showing their origin, molecular weight, and corresponding sequence sources.**

| S No | Protein | Catalog no. (Sigma) | Abbreviation | Origin | Residue | M.W. (Daltons) | Source (Uniprot ID) |
|---|---|---|---|---|---|---|---|
| 1 | Ribonuclease A | R6513 | RNase A | Bovine | 124 | 13690.29 | P61823 (27-150) |
| 2 | Lysozyme | L1667 | LYS | Human | 130 | 14700.67 | P61626 (19-148) |
| 3 | β-casein | C6905 | β-cas | Bovine | 209 | 23583.29 | P02666 (16-224) |
| 4 | Lactoferrin | L4040 | LT | Human | 710 | 78181.95 | P02788 (1-710) |
| 5 | BSA | TC194 (Himedia, India) | BSA | Bovine | 583 | 66432.96 | P02769 (25-607) |
| 6 | Catalase | C40 | CATA | Bovine | 506 | 57583.66 | P00432 (2-527) |
| 7 | β-lactoglobulin | L3908 | β-lac | Bovine | 162 | 18281.21 | P02754 (17-178) |
| 8 | α-lactalbumin | L5385C2 506 | α-lac | Bovine | 123 | 14186.06 | P00711 (20-142) |
| 9 | Myoglobin | M1882 | Mb | Equine heart | 153 | 16951.48 | P68082 (2-154) |
| 10 | Hemoglobin | H7379 | Hb | Human | 141 (α chain)<br>146 (β-chain) | 15126.36 (α chain)<br>15867.22 (β-chain) | P69905 (α-chain) (2-147)<br>P68871 (β-chain) (2-142) |
| 11 | α-amylase | A4551 | α-amy | *Bacillus lichenoformis* | 483 | 55268.17 | P06278 (30-512) |
| 12 | Chymotrypsin | SKU1023 070001 | Chymo | Bovine pancreas | 241 | 25207.64 | P00766 (chain A-1-13, chain B-16-146, chain C-149-245) |
| 13 | α-Synuclein | Expressed and purified | α-Syn | Human | 140 | 14460.16 | P37840 (1-140) |

| | | | | | | | |
|---|---|---|---|---|---|---|---|
| 14 | Tau-F | Expressed and purified | Tau | Human | 441 | 45849.91 | P10636-8 (1-441) |
| 15 | Ovalbumin | A5503 | Alb | Chicken | 385 | 42750.04 | P01012 (2-386) |
| 16 | Ubiquitin | U6253 | Ub | Bovine erythrocytes | 76 | 8530.83 | A0A3Q1M4K3 (1-76) |
| 17 | Cytochrome c | C2506 | Cyt c | Equine | 104 | 11701.55 | P00004 (2-105) |
| 18 | γ-globulin | G5009 | GG | Bovine | * | 150000 | * |
| 19 | Carbonic anhydrase | C3934 | CA | Bovine | * | 30000 | * |

*Notably, sequence information was not available for γ-globulin and carbonic anhydrase.

**Supplementary Table 2. Structural and biophysical characterization of the proteins.** The secondary structure of the proteins was determined by CD spectroscopy. The primary sequence analysis of the proteins was done using IUPred2A[1] to predict the disorder tendency; SMART[2] to analyze the presence of low complexity domains (LCDs) and CatGranule[3] for predicting the propensity for LLPS.

| **S. No.** | **Protein** | **Secondary structure** | **PI** | **Positive charge (P)** | **Negative charge (N)** | **LCD** | **IDR** | **CatGranule Score** |
|---|---|---|---|---|---|---|---|---|
| 1 | RNase A | Helix | 8.64 | 14 | 10 | ✓ | ✓ | -0.93844 |
| 2 | LYS | Helix | 9.28 | 19 | 11 | × | × | -0.0412797 |
| 3 | β-cas | Random coil | 5.13 | 15 | 23 | ✓ | ✓ | -1.15637 |
| 4 | LT | Helix | 8.5 | 90 | 79 | ✓ | ✓ | 0.827097 |
| 5 | BSA | Helix | 5.6 | 82 | 99 | ✓ | × | -0.273782 |
| 6 | CATA | Helix | 6.63 | 58 | 62 | × | ✓ | 0.960901 |
| 7 | β-lac | Helix | 4.83 | 18 | 26 | × | × | -1.22001 |
| 8 | α-lac | Helix | 4.8 | 13 | 20 | ✓ | × | -0.441139 |
| 9 | Mb | Helix | 7.36 | 21 | 21 | × | ✓ | 0.551681 |
| 10 | Hb | Helix | 8.13 | 56 | 54 | × | × | 0.48402 |
| 11 | α-amy | Helix | 6.05 | 50 | 62 | × | ✓ | 1.45616 |
| 12 | Chymo | Random coil | 8.33 | 17 | 14 | × | ✓ | 0.151479 |
| 13 | α-Syn | Random coil | 4.67 | 15 | 24 | ✓ | ✓ | 1.12517 |
| 14 | Tau | Random coil | 8.24 | 58 | 56 | ✓ | ✓ | 1.68922 |
| 15 | Alb | Helix | 5.19 | 35 | 47 | ✓ | ✓ | -0.234022 |
| 16 | Ub | Helix | 6.56 | 11 | 11 | × | ✓ | -0.472928 |
| 17 | Cyt c | Helix | 9.59 | 21 | 12 | × | ✓ | 1.15134 |

**Supplementary Table 3. Different conditions for the protein condensate formation in the presence and absence of PEG-8000 in 20 mM sodium phosphate buffer.** In the presence of PEG-8000, the pH of the solution was maintained at 7.4. In the absence of PEG-8000, different conditions were used for examining protein condensate formation.

| S.No | Protein | $C_{sat}$ (10% PEG-8000, pH 7.4) | Different conditions without PEG-8000 |
|---|---|---|---|
| 1. | GG | 1 μM | 100 μM |
| 2. | LT | 1 μM | 75 μM |
| 3. | CATA | 1 μM | 10 μM and 500 μM NaCl |
| 4. | Chymo | 10 μM | 20 μM at pH 8 |
| 5. | Tau | 10 μM | 100 μM at pH 5 |
| 6. | β-lac | 10 μM | 40 μM and 1M NaCl |
| 7. | β-cas | 20 μM | 30 μM and 1M NaCl |
| 8. | BSA | 20 μM | 100 μM and 1M NaCl |
| 9. | LYS | 100 μM | 100 μM at pH 8 |
| 10. | Alb | 100 μM | 400 μM and 1M NaCl |
| 11. | Hb | 100 μM | 150 μM and 1M NaCl |
| 12. | Cyt c | 100 μM | 200 μM at pH 11 |
| 13. | RNase A | 500 μM | 800 μM at pH 8 |
| 14. | α-Syn | 600 μM | 10 μM at pH 5.5 and 1M NaCl |
| 15. | α-lac | 20 μM | ND |
| 16. | α-amy | 35 μM | ND |
| 17. | CA | 200 μM | ND |
| 18. | Mb | 200 μM | ND |
| 19. | Ub | 1000 μM | ND |

* ND refers to the proteins not determined.

**Supplementary Table 4. Determination of the percentage of secondary structures of the dilute and dense phase of the proteins at LLPS (0 h).** The table shows analysis of a representative dataset of the secondary structure estimation of the dilute and dense phase of proteins at LLPS (0 h) from two independent experiments.

| ***Protein*** | ***α-helix (%)*** | | ***β-sheet (%)*** | | ***Random coil (%)*** | | ***Turn (%)*** | |
|---|---|---|---|---|---|---|---|---|
| | ***Dilute*** | ***Dense*** | ***Dilute*** | ***Dense*** | ***Dilute*** | ***Dense*** | ***Dilute*** | ***Dense*** |
| **LT** | 54 | 55 | 31 | 32 | 0 | 0 | 15 | 13 |
| **GG** | 51 | 56 | 33 | 23 | 0 | 0 | 16 | 20 |
| **CATA** | 56 | 62 | 33 | 29 | 0 | 0 | 11 | 9 |
| **β-lac** | 60 | 56 | 24 | 20 | 0 | 0 | 16 | 23 |
| **Tau** | 18 | 15 | 29 | 31 | 50 | 51 | 3 | 3 |
| **Chymo** | 24 | 25 | 22 | 22 | 40 | 41 | 14 | 12 |
| **BSA** | 43 | 65 | 23 | 17 | 0 | 0 | 34 | 18 |
| **β-cas** | 18 | 16 | 27 | 28 | 50 | 52 | 5 | 4 |
| **α-lac** | 42 | 46 | 18 | 17 | 0 | 0 | 40 | 37 |
| **α-amy** | 46 | 51 | 30 | 29 | 0 | 0 | 24 | 20 |
| **Hb** | 74 | 80 | 17 | 15 | 0 | 0 | 9 | 5 |
| **Alb** | 65 | 69 | 26 | 21 | 0 | 0 | 9 | 10 |
| **LYS** | 56 | 59 | 23 | 16 | 0 | 0 | 21 | 25 |
| **Cyt c** | 64 | 65 | 0 | 0 | 26 | 25 | 10 | 10 |
| **Mb** | 79 | 87 | 21 | 13 | 0 | 0 | 0 | 0 |
| **CA** | 52 | 57 | 34 | 29 | 0 | 0 | 14 | 14 |
| **RNase A** | 56 | 70 | 24 | 19 | 0 | 0 | 20 | 19 |
| **α-Syn** | 0 | 0 | 10 | 10 | 56 | 57 | 34 | 33 |
| **Ub** | 52 | 55 | 28 | 8 | 0 | 0 | 20 | 37 |

## References


1. Mészáros, B., Erdős, G. & Dosztányi, Z. IUPred2A: context-dependent prediction of protein disorder as a function of redox state and protein binding. *Nucleic Acids Res.* **46**, W329–W337 (2018).

2. Letunic, I. & Bork, P. 20 years of the SMART protein domain annotation resource. *Nucleic Acids Res.* **46**, D493–D496 (2017).

3. Bolognesi, B. *et al.* A Concentration-Dependent Liquid Phase Separation Can Cause Toxicity upon Increased Protein Expression. *Cell Rep.* **16**, 222–231 (2016).

4. Xue, B., Dunbrack, R. L., Williams, R. W., Dunker, A. K. & Uversky, V. N. PONDR-FIT: A meta-predictor of intrinsically disordered amino acids. *Biochim. Biophys. Acta - Proteins Proteomics* **1804**, 996–1010 (2010).
