# Supplementary material for: Intermolecular interactions underlie protein/peptide phase separation irrespective of sequence and structure at crowded milieu
Source: Nat Commun. 2023 Oct 4;14:6199. doi: 10.1038/s41467-023-41864-9 (PMC10550955; doi:10.1038/s41467-023-41864-9)
Supplement: Supplementary file 2 — Description of Additional Supplementary Files [file 41467_2023_41864_MOESM2_ESM.pdf]

Description of Additional Supplementary files

Supplementary movie 1: Liquid-liquid phase separation and fusion of Albumin (Alb) Time-lapse movie showing the condensate formation of Alb. The movie shows an increase in condensate size over time due to fusion. The experiment is performed two times with similar observations.

Supplementary movie 2: Liquid-liquid phase separation and fusion of Ubiquitin (Ub) Time-lapse movie showing condensate formation of Ub. The movie shows an increase in condensate size over time due to fusion. The experiment is performed two times with similar observations.
