# Supplementary material for: Intermolecular interactions underlie protein/peptide phase separation irrespective of sequence and structure at crowded milieu
Source: Nat Commun. 2023 Oct 4;14:6199. doi: 10.1038/s41467-023-41864-9 (PMC10550955; doi:10.1038/s41467-023-41864-9)
Supplement: Supplementary file 5 — Reporting Summary [file 41467_2023_41864_MOESM5_ESM.pdf]

nature portfolio

Corresponding author(s): Dr. Samir K. Maji

Last updated by author(s): Sep 15, 2023

Reporting Summary

Nature Portfolio wishes to improve the reproducibility of the work that we publish. This form provides structure for consistency and transparency in reporting. For further information on Nature Portfolio policies, see our Editorial Policies and the Editorial Policy Checklist.

Statistics

For all statistical analyses, confirm that the following items are present in the figure legend, table legend, main text, or Methods section.

n/a	Confirmed	
☐	☒	The exact sample size (*n*) for each experimental group/condition, given as a discrete number and unit of measurement
☐	☒	A statement on whether measurements were taken from distinct samples or whether the same sample was measured repeatedly
☐	☒	The statistical test(s) used AND whether they are one- or two-sided<br>*Only common tests should be described solely by name; describe more complex techniques in the Methods section.*
☒	☐	A description of all covariates tested
☒	☐	A description of any assumptions or corrections, such as tests of normality and adjustment for multiple comparisons
☐	☒	A full description of the statistical parameters including central tendency (e.g. means) or other basic estimates (e.g. regression coefficient) AND variation (e.g. standard deviation) or associated estimates of uncertainty (e.g. confidence intervals)
☐	☒	For null hypothesis testing, the test statistic (e.g. *F*, *t*, *r*) with confidence intervals, effect sizes, degrees of freedom and *P* value noted<br>*Give P values as exact values whenever suitable.*
☒	☐	For Bayesian analysis, information on the choice of priors and Markov chain Monte Carlo settings
☒	☐	For hierarchical and complex designs, identification of the appropriate level for tests and full reporting of outcomes
☐	☒	Estimates of effect sizes (e.g. Cohen's *d*, Pearson's *r*), indicating how they were calculated

Our web collection on statistics for biologists contains articles on many of the points above.

Software and code

Policy information about availability of computer code

Data collection	Confocal microscopy: Instrument-Zeiss Axio-Observer Z1 microscope, Software- Zen 2012;<br>Fluorescence microscopy: Instrument- Leica DMi8 microscope, Software-Leica Application Suite X (LASX);<br>For light scattering: Instrument-Spectrofluorimeter (JASCO FP 8500, USA), Software-SpectraManager V2.0;<br>For Electron Microscopy: Instrument-Transmission electron microscopy (JEOL JEM 2100F, Japan), Software- Gatan microscopy suite;<br>Circular dichroism: Instrument- CD spectrophotometer (JASCO-1500, USA), Software- SpectraManager V2.0;<br>Surface Plasmon Resonance: Instrument- GE Healthcare, BIAcore T200, Software- Biacore T200;<br>Fourier Transform Infrared Spectroscopy: Instrument- Vertex 80 FTIR system equipped with DTGS detector, Software- Opus-65<br>UV-Vis spectroscopy: Instrument- JASCO V650, Japan, Software- SpectraManager V
Data analysis	ImageJ (1.52h), OriginPro 2021, KaleidaGraph (v4.03), GraphPad Prism 8, SR0 v8.0724 (B724)

For manuscripts utilizing custom algorithms or software that are central to the research but not yet described in published literature, software must be made available to editors and reviewers. We strongly encourage code deposition in a community repository (e.g. GitHub). See the Nature Portfolio guidelines for submitting code & software for further information.

Data

Policy information about availability of data

All manuscripts must include a data availability statement. This statement should provide the following information, where applicable:

- Accession codes, unique identifiers, or web links for publicly available datasets
- A description of any restrictions on data availability
- For clinical datasets or third party data, please ensure that the statement adheres to our policy

The authors declare that all the data supporting the findings of this study are available within the paper and its Supplementary Information text. All the data analysis were performed using published tools and packages and has been cited in the paper and supplementary information text. Source data are provided with this paper.PDB ID: 1B0L (https://doi.org/10.2210/pdb1B0L/pdb), 1QG5 (https://doi.org/10.2210/pdb1QG5/pdb), 3V03 (https://doi.org/10.2210/pdb3V03/pdb), 1REX (https://doi.org/10.2210/pdb1REX/pdb), 1FS3 (https://doi.org/10.2210/pdb1FS3/pdb), 8F2H (https://doi.org/10.2210/pdb8F2H/pdb), 4LB2 (https://doi.org/10.2210/pdb4LB2/pdb). PED ID: PED00017e001(https://proteinensemble.org/entries/PED00017).

Human research participants

Policy information about studies involving human research participants and Sex and Gender in Research.

Reporting on sex and gender	NA
Population characteristics	NA
Recruitment	NA
Ethics oversight	NA

Note that full information on the approval of the study protocol must also be provided in the manuscript.

Field-specific reporting

Please select the one below that is the best fit for your research. If you are not sure, read the appropriate sections before making your selection.

☒ Life sciences ☐ Behavioural & social sciences ☐ Ecological, evolutionary & environmental sciences

For a reference copy of the document with all sections, see nature.com/documents/nr-reporting-summary-flat.pdf

Life sciences study design

All studies must disclose on these points even when the disclosure is negative.

Sample size	Sample size was based on the reproducibility of the data from independent experiments (n=2 or more) to get statistically significant means and standard deviations. For CD, FTIR, SPR and TEM experiments were performed twice and FRAP assay , three independent biological replicates were performed. Samples were analyzed and corresponding mean and standard deviations/ errors were calculated for their statistical significance (wherever applicable) and were reported in the main and supplementary manuscript. P-values are reported in the respective figure legends and source data file.
Data exclusions	No data were excluded during the analysis.
Replication	All experiments were repeated at least twice/thrice. The number of replicates used in the individual experiments are mentioned in the respective figure legends. All the replicates were successfully reproducible and showed similar results.
Randomization	Randomization is not relevant in our study as we performed the assays in vitro and the samples/data were not taken from any existing distribution
Blinding	Investigators were not blinded to the studies due to the inability to administer relevant treatments during the course of experiment if blinded.

Reporting for specific materials, systems and methods

We require information from authors about some types of materials, experimental systems and methods used in many studies. Here, indicate whether each material, system or method listed is relevant to your study. If you are not sure if a list item applies to your research, read the appropriate section before selecting a response.

Materials & experimental systems

n/a	Involved in the study	
☒	☐	Antibodies
☐	☒	Eukaryotic cell lines
☒	☐	Palaeontology and archaeology
☒	☐	Animals and other organisms
☒	☐	Clinical data
☒	☐	Dual use research of concern

Methods

n/a	Involved in the study	
☒	☐	ChIP-seq
☒	☐	Flow cytometry
☒	☐	MRI-based neuroimaging

Eukaryotic cell lines

Policy information about cell lines and Sex and Gender in Research

Cell line source(s)	Authenticated HeLa was procured from NCCS cell repository, Pune, India
Authentication	NCCS cell repository, Pune authenticated the cell line by STR profiling using AmpFISTR Indentifiler Plus PCR amplification kit from Applied Biosystems
Mycoplasma contamination	No mycoplasma contamination with the cells as tested by NCCS cell repository, Pune, India.
Commonly misidentified lines (See ICLAC register)	No commonly misidentified cell lines were used in this study.

March 2021
